# The EHMT2-MBLAC2 axis suppresses ribosomal DNA transcription in response to nucleolar DNA damage

**DOI:** 10.1038/s41419-026-08616-1

**Published:** 2026-03-18

**Authors:** Chenyue Wang, Qiutian Lu, Lianbao Cao, Simeng Zeng, Zihan Gao, Yinglong Yang, Xiaowen Liu, Shanshan Gao, Chao Dong

**Affiliations:** 1 Department of Occupational and Environmental Health, School of Public Health, Cheeloo College of Medicine, Shandong University, Jinan, Shandong China; 2 Department of Gynecologic Oncology, Shandong Cancer Hospital and Institute, Shandong First Medical University and Shandong Academy of Medical Sciences, Jinan, Shandong China; 3 Department of Gastroenterology, The First Affiliated Hospital of Shandong First Medical University & Shandong Provincial Qianfoshan Hospital, Jinan, Shandong China; 4 Present Address: Futaste Pharmaceutical Co. Ltd., Dezhou, Shandong China

**Keywords:** DNA damage and repair, Molecular biology

## Abstract

The induction of DNA double-strand breaks (DSBs) within actively transcribed ribosomal DNA (rDNA) arrays triggers transcriptional suppression and drives nucleolar reorganization, including the formation of nucleolar caps that facilitate the engagement of DSBs with canonical DSB signaling and repair proteins. Although these nucleolar responses are critical for rDNA stability, the components that orchestrate these responses remain unclear. In this study, we identified euchromatic histone-lysine N-methyltransferase 2 (EHMT2) as a novel regulator that is essential for rDNA DSB-induced transcriptional suppression, while functioning independently of ATM-mediated nucleolar responses. We found that EHMT2 is required for the repair of rDNA DSBs and the maintenance of rDNA stability, and its deficiency can result in cellular hypersensitivity to rDNA DSBs. Global proteomic analysis revealed that EHMT2 interacts with MBLAC2 to repress rDNA transcription upon rDNA DSBs. The depletion of EHMT2 or MBLAC2 sensitized colorectal cancer cells to ribosomal stress. Furthermore, we uncovered that EHMT2 promotes colorectal tumorigenesis, revealing a novel mechanistic link between rDNA transcriptional regulation and tumor promotion. Together, our findings established the EHMT2-MBLAC2 axis as a pivotal regulator of mammalian rDNA DSB-induced transcriptional silencing that coordinates rDNA DSB repair and the maintenance of rDNA integrity during nucleolar damage.

## Introduction

Ribosomal DNA (rDNA) resides in the nucleolus, which is a dynamic non-membrane organelle and is endowed with high transcription frequency and tandem repeats; hence, rDNA is susceptible to DNA double-strand breaks (DSBs) [1]. Failure to maintain rDNA stability can lead to rDNA copy number variations and disrupt cellular homeostasis [2, 3]. rDNA instability has been implicated in the pathogenesis of various human diseases, including cancers [4–6]. Therefore, DSBs introduced into rDNA arrays must be accurately and efficiently repaired to preserve their stability.

DSBs are widely regarded as one of the most deleterious forms of DNA damage, and rDNA DSBs can elicit specialized nucleolar DNA damage responses (DDRs), including extensive nucleolar structure reorganization, the translocation of damaged rDNA inside the nucleolus to the nucleolar periphery, formation of nucleolar caps, and transcriptional suppression of RNA polymerase I (RNAPI) with phase separation [7–11]. rDNA DSBs induce the formation of nucleolar caps that act as hubs for DSB signaling and repair proteins, thus facilitating repair and preserving rDNA integrity [7, 12].

The prompt and transient suppression of RNAPI transcription following rDNA DSBs is orchestrated by a complex network of factors, encompassing the DDR factors ataxia-telangiectasia-mutated (ATM) and ATM and RAD3-related (ATR), human silencing hub (HUSH) complex, Nijmegen breakage syndrome protein 1 (NBS1), the kinases mammalian STE20-like kinase 2 (MST2) and dual specificity tyrosine phosphorylation regulated kinase 1B (DYRK1B) [7, 13–16]. In this context, the master DDR kinases ATM and ATR play pivotal roles in both rDNA DSB-induced transcriptional silencing and nucleolar segregation to orchestrate nucleolar DDRs [7, 8, 17]. The HUSH complex drives H3K9me3 deposition on damaged rDNA, thereby mediating transcriptional silencing and promoting DSB repair [13]. Similarly, the MST2 kinase drives rDNA transcriptional shutdown via chromatin remodeling, specifically by phosphorylating nucleolar histone H2B at serine 14 (H2BS14p), highlighting the regulatory impact of chromatin modifications on the nucleolar damage response. Additionally, the accumulation of Treacle-dependent NBS1 and TOPBP1 in the nucleoli leads to the repression of ribosomal RNA synthesis and nucleolar segregation upon rDNA DSB formation [14, 17]. Recently, we identified a DYRK1B-dependent pathway that suppresses RNAPI transcription in response to rDNA DSBs [18]. Although these studies have advanced our understanding of rDNA transcriptional repression, the repair of rDNA DSBs remains poorly understood. While the precise mechanisms of rDNA DSB repair remain contentious, emerging evidence indicates a preference for the non-homologous end joining (NHEJ) pathway in the repair of nucleolar rDNA breaks [7, 19]. Contrary to the proposed model of high-fidelity homologous recombination (HR)-mediated repair of rDNA DSBs in nucleolar caps throughout the cell cycle [12, 13], evidence supporting a DNA-PK-dependent NHEJ pathway for lesions at the nucleolar periphery has been reported [7]. However, the precise regulatory circuitry that couples DSB-induced rDNA transcriptional silencing with the DNA repair machinery to safeguard genome stability remains unclear.

EHMT2 (also known as G9a), a member of the histone lysine methyltransferase family, catalyzes histone 3 lysine 9 (H3K9) monomethylation and dimethylation in euchromatin and plays critical roles in promoting human malignancies and gene suppression [20]. Chromatin-enriched EHMT2 directly participates in DDR by coordinating with RPA to promote the deposition of RPA and Rad51 at DSB sites [21]. EHMT2 catalyzes the methylation of MDC1 at lysine 45, a critical posttranslational modification that facilitates the recruitment and accumulation of ATM at DNA DSBs [22]. Moreover, localization of EHMT2 to DNA damage sites is dependent on ATM-mediated phosphorylation, and its catalytic activity is indispensable for the early accumulation of 53BP1 and BRCA1 [23]. Our recent findings revealed that DYRK1B phosphorylates EHMT2 and promotes its accumulation in DSB-flanking chromatin to facilitate DSB-induced RNAPI transcriptional silencing and coordinates DSB repair activity on the transcribed chromatin [15]. Notably, despite the established role of EHMT2 in DDR, its function in the context of the nucleolar DNA damage response is poorly understood.

In the present study, we aimed to report an EHMT2-dependent signaling pathway that regulates nucleolar DDRs, specifically suppressing RNAPI-mediated transcription in response to rDNA DSBs. Our results established EHMT2 as an integral component of the nucleolar DDR, which is essential for the repair of rDNA DSBs and for the preservation of rDNA stability and cell viability following the induction of rDNA DSBs. Global proteomic analysis of EHMT2 substrates identified MBLAC2, the function of which remains elusive in DDR, as a key regulator of rDNA DSB-induced transcriptional suppression. Furthermore, we defined the EHMT2-MBLAC2 signaling axis as a pathogenic driver in human cancers, and demonstrated its role in promoting colorectal tumorigenesis. Together, our findings revealed a key role of the EHMT2-MBLAC2 signaling axis in rDNA DSB-induced transcriptional suppression and colorectal tumorigenesis.

## Results

### EHMT2 promotes rDNA DSB-induced transcriptional suppression

**Fig. 1:**
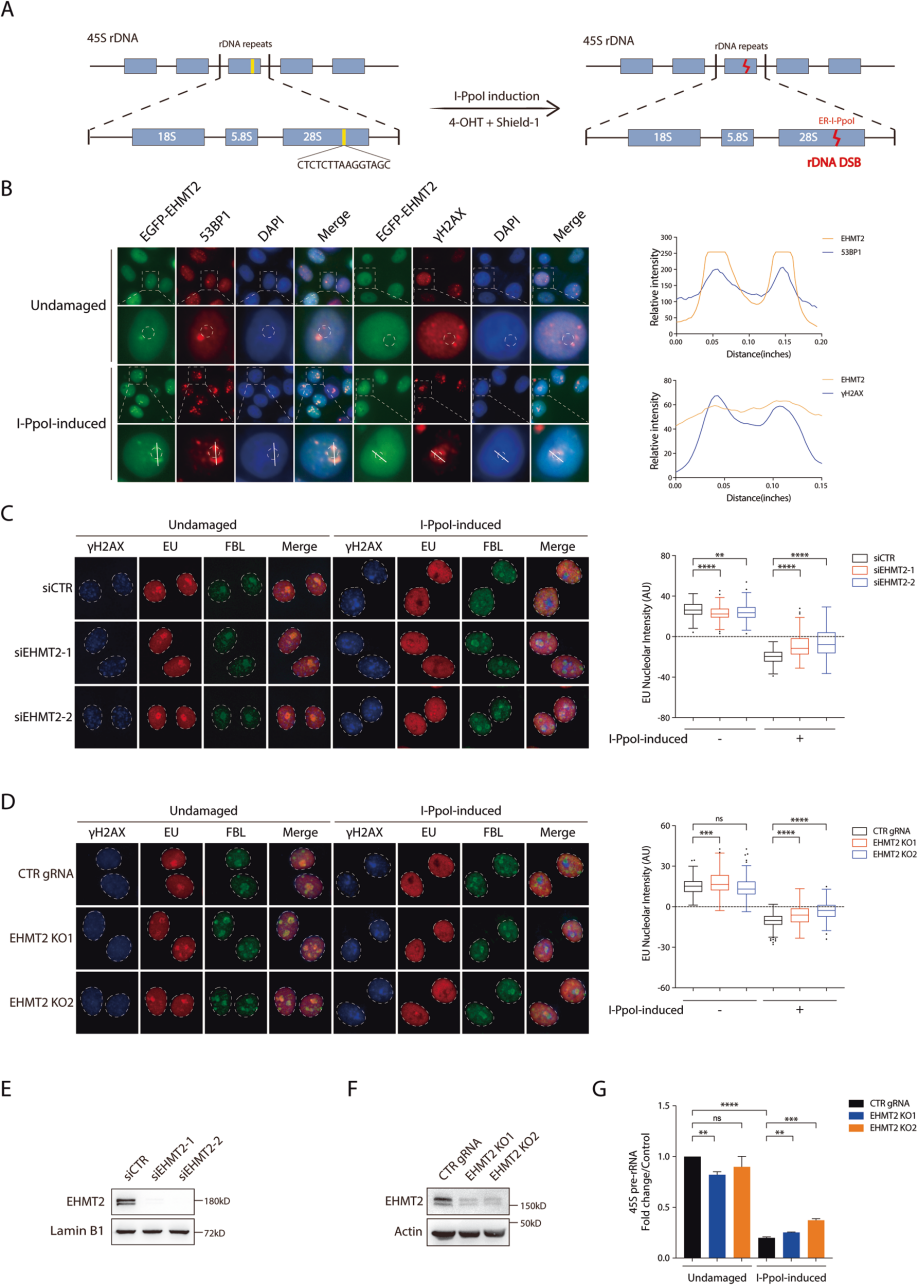
EHMT2 promotes rDNA DSB-induced transcriptional suppression. **A** Schematic illustration of 45S rDNA repeats with I-*Ppo*I endonuclease targeting sequence. Cells were treated with 4-OHT and Shield-1 to induce the expression of I-*Ppo*I endonuclease leading to the introduction of DSBs into 28S rDNA arrays and the subsequent suppression of rDNA transcription. **B** The formation of EHMT2 nucleolar caps in HeLa I-*Ppo*I cells following rDNA DSBs induction. HeLa *I-Ppo*I cells transfected with EGFP-EHMT2 plasmid were treated with 1 μM Shield-1 and 2 μM 4-OHT for 4 h to induce rDNA DSBs before fixation. Fixed cells were labeled with 53BP1 or γH2AX. Nuclei were counterstained with DAPI. Enlarged images depict the details of the dashed circle labeled nuclei. Quantification of relative signal intensities of EGFP-EHMT2, 53BP1 and γH2AX caps was performed by ImageJ using the ROI tool. The white lines in the enlargements of the representative images indicate the lines for quantification. The edge of the indicated nucleolar caps was labeled with dotted circle. **C** Analysis of nucleolar transcription activity by 5-Ethynyl Uridine (EU) incorporation assay in EHMT2-deficient HeLa I-*Ppo*I cells following rDNA DSBs induction. Cells transfected with control (siCTR) or two independent EHMT2-targeted siRNAs were induced for rDNA DSBs for 4 h. Cells were subsequently cultured in medium supplemented with EU for 1 h before fixation. Fixed cells were labeled with EU, nucleolar marker Fibrillarin (FBL) and DSB marker γH2AX. Quantification of relative EU nucleolar intensity from three independent experiments is shown in Tukey boxplots. **D** HeLa I-*Ppo*I cells transduced with control gRNA (CTR gRNA) and two EHMT2 gRNAs (EHMT2 KO1 and EHMT2 KO2) were subjected to EU incorporation assay as described in (**C**). Quantification of relative EU nucleolar intensity from three independent experiments is shown in Tukey boxplots. **E** siRNA-mediated EHMT2 knockdown efficiency was measured by Western blotting. **F** CRISPR/Cas9-mediated EHMT2 knockout efficiency was measured by immunoblotting. **G** HeLa I-*Ppo*I cells transduced with control gRNA (CTR gRNA) and two EHMT2 gRNAs (EHMT2 KO1 and EHMT2 KO2) were treated with 1 μM Shield-1 and 2 μM 4-OHT to induce rDNA DSBs for 3 h, and left to rest on ice for 10 minutes, then were further treated with 1 μM of Shield-1 and 2 μM of 4-OHT solution for 1 h. Quantification of 45S pre-rRNA fold change is shown and data were from four independent experiments. Bars represent mean ± SEM; ns not significant; ^**^P < 0.01; ^***^P < 0.001; ^****^P < 0.0001.

To investigate the role of EHMT2 in rDNA DSB-induced transcriptional suppression, we employed a previously established endonuclease I-*Ppo*I cell system in which I-*Ppo*I could be induced by the presence of 4-hydroxytamoxifen (4-OHT) and Shield-1 to generate a targeted rDNA DSB within 28S rDNA repeats [7, 18, 24] (Fig. 1A). We first validated the I-*Ppo*I-inducible stable cells and confirmed that rDNA DSBs could be induced upon the addition of 4-OHT and Shield-1 by the robust observation of γH2AX and 53BP1 nucleolar cap formation in more than 80% of the cells (Supplementary Fig. S1A, B). Consistent with previous studies, marked colocalization of DSB markers γH2AX/53BP1 with nucleolar markers such as fibrillarin (FBL) or upstream binding transcription factor (UBF) were observed at the nucleolar periphery (Supplementary Fig. S1A, B), thereby acting as spatial landmarks to effectively visualize rDNA DSBs and demarcate nucleolar boundaries in response to I-*Ppo*I-induced rDNA damage [12, 18, 25]. We next assessed the recruitment of EHMT2 to the nucleolar periphery in response to rDNA breaks using the I-*Ppo*I-based platform and found that EGFP-EHMT2 accumulated at the nucleolar periphery upon I-*Ppo*I induction, colocalizing with γH2AX and 53BP1 nucleolar caps, as determined by immunofluorescent microscopy (Fig. 1B). Interestingly, we found that the dynamics of γH2AX and 53BP1 nucleolar cap formation were significantly compromised in EHMT2-deficient cells following I-*Ppo*I-induced rDNA DSBs (Supplementary Fig. S1C). These observations indicated the requirement of EHMT2 for a proper rDNA DSB response. To assess the role of EHMT2 in RNAPI-driven transcription upon rDNA DSB formation, we performed 5-ethynyl uridine (EU) incorporation assay to track nascent rRNA transcription by integrating the uridine analog EU during rDNA transcription. Sustained nucleolar EU signals were observed in EHMT2-deficient cells upon I-*Ppo*I induction (Fig. 1C and Supplementary Fig. S1D), suggesting that EHMT2 is required for the efficient suppression of transcription at rDNA DSBs. Consistent with these findings, a similar impairment in transcriptional repression was confirmed in CRISPR-Cas9-mediated EHMT2-knockout (KO) I-*Ppo*I stable cells (Fig. 1D and Supplementary Fig. S1E). The siRNA-mediated EHMT2 knockdown and CRISPR-Cas9-mediated EHMT2 KO efficiencies were examined by immunoblotting (Fig. 1E, F).

To corroborate the observations derived from EU incorporation assays, we assessed the nascent rRNA synthesis by the quantification of 45S precursor ribosomal RNA (45S pre-rRNA) transcript using quantitative real-time polymerase chain reaction (qPCR). Consistent with the role of EHMT2 in promoting rDNA damage-induced transcriptional repression, its inhibition significantly attenuated 45S pre-rRNA suppression (Fig. 1G). To ascertain whether the change in 45S pre-rRNA levels reflects transcription rather than altered RNA processing, we employed nuclear run-on assays as a direct measure of transcriptional activity and found that EHMT2 deficiency reproducibly compromised rDNA damage-induced transcriptional repression (Supplementary Fig. S1F).

Taken together, these observations suggested that EHMT2 facilitates nucleolar transcriptional suppression following I-*Ppo*I-induced rDNA DSBs.

### Methyltransferase activity is required for EHMT2-mediated rDNA DSB-induced transcriptional suppression

**Fig. 2:**
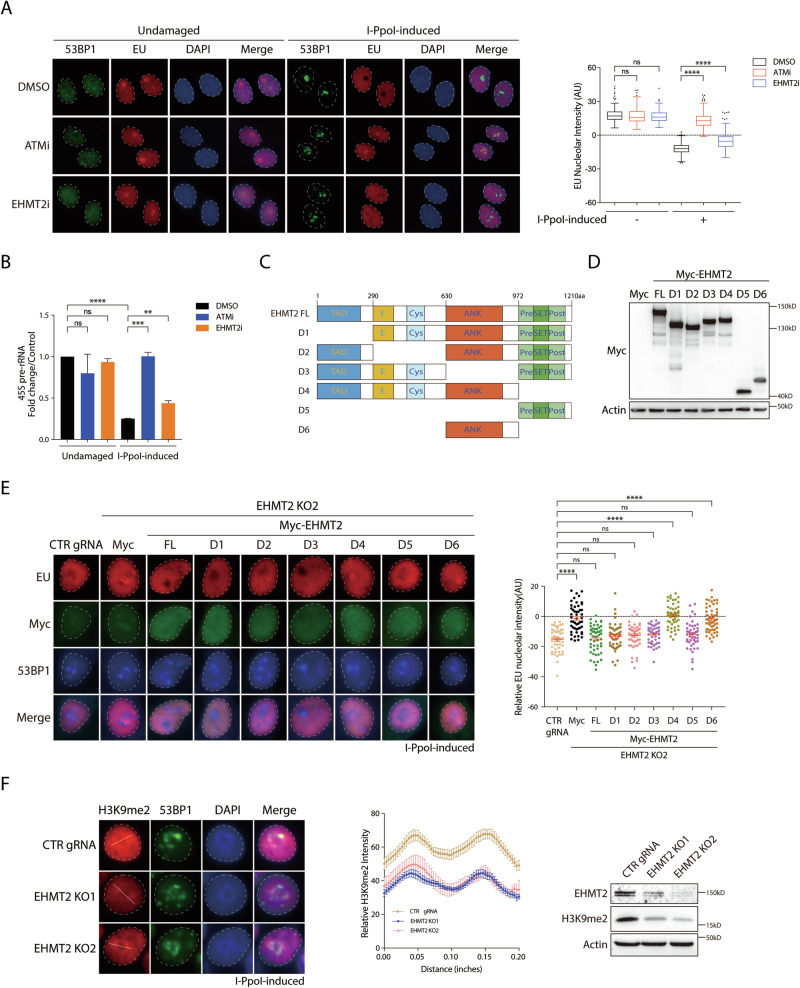
EHMT2 mediates rDNA DSB-induced transcriptional suppression via its methyltransferase activity. **A** Analysis of nucleolar transcription activity by EU incorporation assay in EHMT2-inactivated HeLa I-*Ppo*I cells following rDNA DSBs induction. HeLa I-*Ppo*I cells were pre-treated with ATM inhibitor (ATMi; KU-55933) or EHMT2 inhibitor (EHMT2i; UNC0638) for 1 h prior to rDNA DSBs induction and induced for another 4 h-induction of rDNA DSBs. EU nucleolar intensity was subsequently determined by EU incorporation assay. At least 200 cells exhibiting well-circumscribed nucleoli were quantitatively assessed across minimally three independent experiments. Quantification of relative EU nucleolar intensity is shown in Tukey boxplots. **B** Fold change of 45S pre-rRNA in HeLa I-*Ppo*I cells pre-treated with ATM inhibitor (ATMi; KU-55933) or EHMT2 inhibitor (EHMT2i; UNC0638) after I-*Ppo*I induction was determined by RT-qPCR. Quantification of 45S pre-rRNA fold change was from four independent experiments. **C** Schematic illustration showing domain structure of EHMT2 protein and its truncated mutants. **D** Protein expression of the indicated Myc-tagged EHMT2 truncated mutants was measured by Western blotting. **E** HeLa I-*Ppo*I cells transduced with EHMT2 gRNA2 were reconstituted with the indicated Myc-tagged EHMT2 truncated mutants. Cells were subjected to EU incorporation assay after I-*Ppo*I induction. Dashed circle shows the margins of the representative nucleoli. Quantification of relative EU nucleolar intensity was derived from three independent experiments and is shown in Scatter plot. **F** H3K9me2 nucleolar intensity was determined in EHMT2-deficient HeLa I-*Ppo*I cells. EHMT2-KO I-*Ppo*I cells were subjected to immunofluorescence and were labeled with H3K9me2 and 53BP1 after rDNA DSBs induction. Nuclei were counterstained with DAPI. Dashed circle shows the margins of the nucleoli. The white lines in the representative images indicate the lines for quantification. Quantification of relative signal intensities of H3K9me2 in the representative images was shown. The protein levels of H3K9me2 were measured by immunoblotting. Bars represent mean ± SEM; ns not significant; ^**^P < 0.01; ^***^P < 0.001; ^****^P < 0.0001.

As EHMT2 is a methyltransferase, we examined whether its catalytic activity is essential for EHMT2-mediated rDNA DSB-induced transcriptional suppression. Using the EU incorporation assay, we found that cells pretreated with the EHMT2 inhibitor (EHMT2i, UNC0638), similar to those treated with the ATM inhibitor (ATMi, KU55933), failed to suppress nucleolar transcription upon I-*Ppo*I induction (Fig. 2A). In agreement with the EU staining data, quantification of 45S pre-rRNA transcripts revealed a significant increase in EHMT2 inhibition (Fig. 2B), indicating that EHMT2 inhibition led to a compromised suppression of 45S pre-rRNA synthesis. These observations showed that pharmaceutical inhibition of EHMT2 leads to a defect in rDNA DSB-induced transcriptional silencing, suggesting that the methyltransferase activity of EHMT2 is necessary for suppressing RNAPI transcription upon I-*Ppo*I induction.

To further determine the methyltransferase activity of EHMT2 in suppressing rDNA transcription at rDNA DSBs, we generated a panel of EHMT2-truncated mutants (Fig. 2C) and verified their protein expression via immunoblot analysis (Fig. 2D). Together with the EU and RT-qPCR data, we found that the re-expression of wild-type EHMT2, but not its SET domain-deleted mutants (D4 and D6), in EHMT2-inactivated cells restored nucleolar transcriptional silencing following I-*Ppo*I induction (Fig. 2E). Consistent with the requirement of EHMT2 catalytic activity in rDNA DSB-induced transcriptional repression, the EHMT2 SET domain (D5) was sufficient to suppress transcription in response to rDNA DSBs (Fig. 2E). As the primary methyltransferase for the dimethylation of histone H3 at lysine 9 (H3K9me2), EHMT2 was found to be essential for the deposition of this mark at rDNA break-induced nucleolar caps, as depletion of EHMT2 impaired the formation of H3K9me2-marked nucleolar caps following rDNA DSBs induction (Fig. 2F), thereby indicating the requirement of its catalytic activity and EHMT2-mediated H3K9me2 deposition in transcriptional repression.

DYRK1B, a key regulator of rDNA damage-induced silencing, phosphorylates EHMT2 at threonine 555 (T555) to drive the recruitment and deposition of EHMT2 at DSBs sites [15]. Here, we investigated whether the functional requirement of EHMT2 in rDNA DSB-induced transcriptional silencing depends on its phosphorylation by DYRK1B. We generated EHMT2 phospho-mutants (T555A and T555D) and investigated their ability to suppress nucleolar transcription after rDNA DSB formation (Supplementary Fig. S2A, B). Interestingly, the phospho-mimicking EHMT2 T555D mutant displayed robust accumulation at the periphery of 53BP1-containing nucleolar caps, suggesting that DYRK1B facilitates EHMT2 docking to rDNA DSB-induced nucleolar caps by phosphorylating EHMT2 at the T555 site (Supplementary Fig. S2C), and EHMT2 could function downstream of DYRK1B to repress the transcription upon rDNA DSBs. However, neither the non-phosphorylated (T555A) nor the phospho-mimetic (T555D) EHMT2 mutant significantly suppressed nucleolar transcription following rDNA DSB formation (Supplementary Fig. S2C), indicating that the function of EHMT2 in transcriptional silencing is uncoupled from its DYRK1B-mediated phosphorylation and nucleolar cap deposition. Collectively, these data suggested that DYRK1B orchestrates EHMT2 accumulation at rDNA DSBs through T555 phosphorylation and that methyltransferase activity is required for EHMT2-mediated rDNA DSB-induced transcriptional repression.

### EHMT2-mediated rDNA DSB-induced transcriptional suppression is independent of nucleolar segregation

**Fig. 3:**
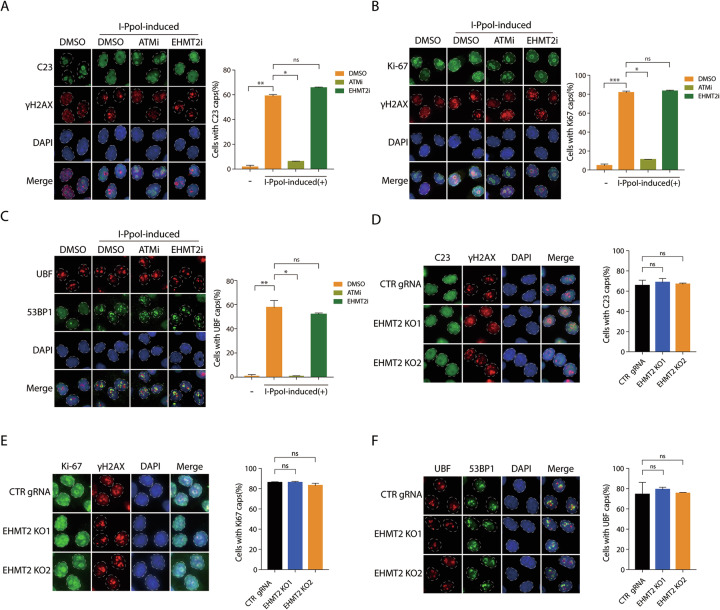
EHMT2-mediated nucleolar transcriptional silencing is independent of nucleolar segregation. **A–C** HeLa I-*Ppo*I cells were pre-treated with ATM inhibitor (ATMi; KU-55933) or EHMT2 inhibitor (EHMT2i; UNC0638) for 1 h prior to I-*Ppo*I induction for 4 h. After fixation, cells were subjected to immunofluorescence with anti-C23 (**A**), anti-Ki-67 (**B**), anti-UBF (**C**) or anti-γH2AX antibodies, respectively. The dashed circles outline margins of the nuclei. Percentages of cells with the indicated protein-containing nucleolar caps were quantified from three experiments. **D**–**F** EHMT2-KO HeLa I-*Ppo*I cells were processed for immunofluorescence as described in (**A**–**C**). Cells were labeled with anti-C23 (**D**), anti-Ki-67 (**E**), anti-UBF (**F**) or anti-γH2AX antibodies, respectively. Nuclei were counterstained with DAPI. Percentages of cells with the nucleolar caps were quantified from three experiments. Bars represent mean ± SEM; ns not significant; ^*^P < 0.05; ^**^P < 0.01; ^***^P < 0.001.

While ATM is the central mediator of rDNA DSB-induced transcriptional suppression and nucleolar reorganization, we had established that DYRK1B facilitates nucleolar transcriptional silencing at rDNA DSBs through a distinct, nucleolar reorganization-independent pathway [7, 18]. Therefore, we assessed whether EHMT2 elicits nucleolar segregation while simultaneously repressing rDNA transcription following I-*Ppo*I induction. To this end, we examined the distributions of nucleolar proteins C23, Ki-67, and UBF in EHMT2-inactivated HeLa I-*Ppo*I cells after rDNA DSB induction. Consistent with previous findings, ATM orchestrated the suppression of RNAPI-driven transcription concurrent with a profound nucleolar reorganization, which included the relocalization of key nucleolar markers (C23, Ki-67, and UBF) to the nucleolar interior (Fig. 3A–C). Notably, we found that pharmaceutical inhibition of EHMT2 did not noticeably alter the redistribution of C23 (Fig. 3A), Ki-67 (Fig. 3B), or UBF (Fig. 3C) in response to I-*Ppo*I-induced rDNA DSBs, which is distinct from ATM-driven reorganization. Interestingly, these findings were consistent with the observations in DYRK1B-inactivated cells, suggesting that EHMT2 resembles DYRK1B and may specifically facilitate rDNA DSB-induced transcriptional silencing, regardless of nucleolar segregation. Similarly, CRISPR/Cas9-mediated EHMT2 KO did not noticeably affect the redistributions of C23 (Fig. 3D), Ki-67 (Fig. 3E), or UBF (Fig. 3F) in the I-*Ppo*I-induced nucleolar caps. Moreover, neither chemical inhibition (Fig. 3A–C) nor CRISPR/Cas9-mediated EHMT2 inactivation (Fig. 3D–F) disrupted the redistribution of γH2AX or 53BP1 following rDNA DSB formation. These data suggested that EHMT2-mediated rDNA DSB-induced transcriptional suppression is independent of nucleolar reorganization, and that its mechanism of action parallels that of DYRK1B, distinct from that of ATM.

### EHMT2 promotes rDNA DSB responses

**Fig. 4:**
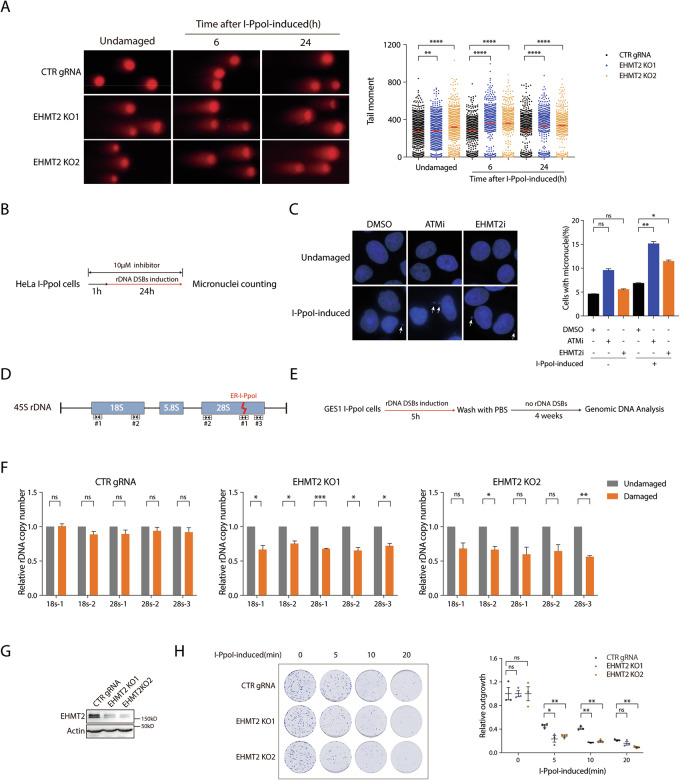
EHMT2 promotes rDNA DSB responses. **A** HeLa I-*Ppo*I cells lenti-virally transduced with control gRNA (CTR gRNA) and EHMT2-targeting gRNAs (EHMT2 KO1 and EHMT2 KO2) were induced with Shield-1 and 4-OHT for 4 h to introduce DSBs into rDNAs. Cells were subjected to neutral comet assay at 6 h and 24 h after recovery from 4 h-rDNA DSBs induction. Relative tail moment from three independent experiments were analyzed and plotted. **B** The schematic illustration depicts the workflow of micronuclei counting in response to rDNA DSBs. **C** Representative images depict the micronuclei derived from undamaged and I-*Ppo*I induced cells treated with ATM or EHMT2 inhibitors. Quantification shows the percentage of cells with micronuclei. Data from three independent experiments were quantified. White arrowheads indicate the locations of micronuclei. **D** Schematic diagram represents 45S rDNA repeats. I-*Ppo*I targeted sequence is shown. The labels and the coverage indicated by the arrowheads suggest the primers used in the following quantification of rDNA copy number in I-*Ppo*I survival cells. **E** The flow diagram describes the procedure of quantification of rDNA copy number in I-*Ppo*I survivor cells. **F** The relative rDNA copy number in EHMT2-deficient GES1 I-*Ppo*I survival cells was measured and quantified as depicted in (**G**). Data were derived from three independent experiments. **G** CRISPR–Cas9-mediated EHMT2 knockout (KO) efficiency was validated by Western blotting. **H** Clonogenic survival of EHMT2 KO HeLa I-*Ppo*I cells were quantified upon I-*Ppo*I activation. Briefly, rDNA DSBs in cells were induced with Shield-1 and 4-OHT for 5, 10, or 20 min. Cells were then washed with PBS twice and were cultured to grow for ten days before subjecting to Coomassie blue staining. Bars represent mean ± SEM; ns not significant; ^*^P < 0.05; ^**^P < 0.01; ^***^P < 0.001; ^****^P < 0.0001.

Transcriptional silencing at DSB sites is well established as imperative for faithful DSB repair. The prevailing model holds that the transient transcriptional silencing near DSBs is critical both to avoid conflicts with the DSB repair machinery and to suppress the generation of aberrant truncated RNAs with potentially deleterious functions when DSBs occur within a gene body [26, 27]. Recent studies suggest that silencing factors implicated in DSB-induced transcriptional repression may serve as integral components of the DNA repair apparatus [7, 15, 18, 28]. Given the role of EHMT2 in modulating transcription at rDNA DSBs and the critical link between repair failure and rDNA instability, we next investigated its role in the rDNA DSB repair process. To assess the impact of EHMT2 loss on rDNA DSB repair, we used a neutral comet assay to measure the DNA tail moment in EHMT2-inactivated cells after a recovery period following I-*Ppo*I induction. Sustained DNA damage was observed in EHMT2-inactivated cells after rDNA DSB induction, suggesting that the loss of EHMT2 led to defects in rDNA DSB repair (Fig. 4A). To further determine the repair pathways involved in EHMT2-mediated rDNA DSB repair, we examined the recruitment of HR repair factors (Rad51 and BRCA1) and NHEJ factors (Ku80 and DNA-PKcs) to the nucleolar caps of EHMT2 KO cells following I-*Ppo*I-induced DSBs. Interestingly, we found that the recruitment of HR repair factors Rad51 and BRCA1 to the nucleolar caps was not noticeably compromised upon EHMT2 loss (Supplementary Fig. S3A, B), whereas deposition of the NHEJ proteins (Ku80 and DNA-PKcs) to the nucleolar caps was significantly reduced (Supplementary Fig. S3C, D), suggesting that EHMT2 possibly facilitated rDNA DSB repair primarily via the NHEJ repair machinery.

To determine whether the rDNA DSB repair defect caused by EHMT2 loss compromises rDNA stability, we assessed genomic instability by measuring micronucleus formation (Fig. 4B). High frequencies of micronuclei were observed in EHMT2-inhibited cells upon I-*Ppo*I induction, indicating that EHMT2-mediated rDNA DSB repair is critical for preserving rDNA stability (Fig. 4C). Consistent results were observed in EHMT2-KO cells following I-*Ppo*I-induced rDNA DSBs (Supplementary Fig. S3E). To more accurately decipher how EHMT2 deficiency induces genetic instability in rDNA arrays, we next determined the abundance of rDNA copy numbers in EHMT2-KO cells. The rDNA copy number was quantified using RT-qPCR after a recovery period following rDNA DSB induction, as illustrated schematically (Fig. 4D, E). Consistent with the role of EHMT2 in preserving rDNA stability, we found that EHMT2 deficiency led to a significant decrease in rDNA copy number in the I-*Ppo*I cells recovered from rDNA DSBs (Fig. 4F, G), further suggesting that EHMT2 preserved rDNA stability in response to rDNA DSBs. Given the observed defects in rDNA repair and stability following EHMT2 loss, we examined whether the genomic instability compromised cell survival following rDNA DSB induction. By performing clonogenic survival assays, we found that EHMT2 loss rendered the cells hypersensitive to I-*Ppo*I induced rDNA DSBs (Fig. 4H), underscoring its critical role in promoting cell viability following rDNA damage. Taken together, these data suggested that EHMT2 is a critical component in rDNA DSB repair and in the maintenance of rDNA stability and cell survival following rDNA DSBs.

### Proteomic analysis of EHMT2 substrates identifies rDNA DSB-induced transcriptional suppression factors

**Fig. 5:**
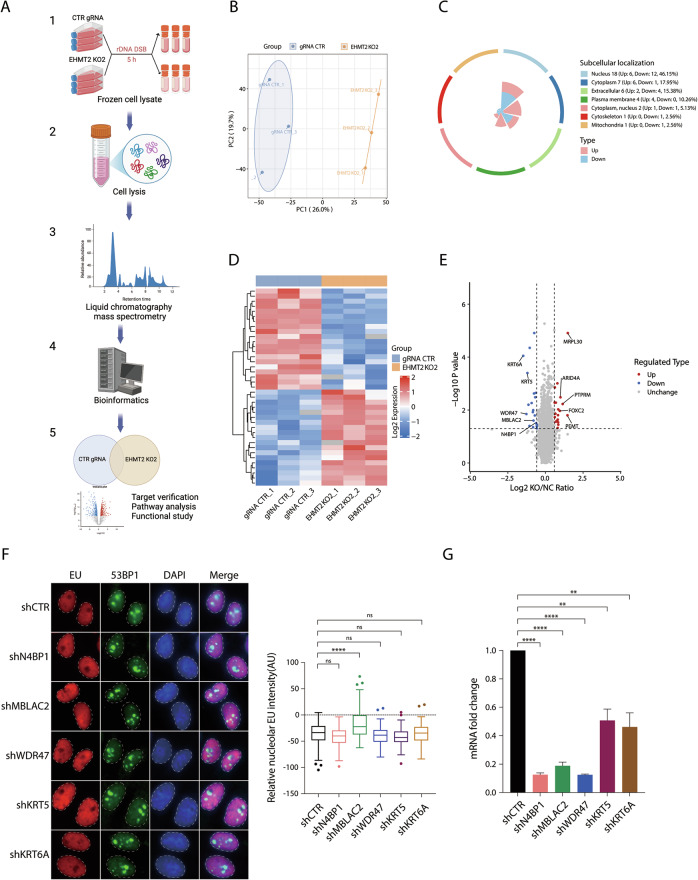
Proteomic analysis of EHMT2 substrate identifies novel factors that regulate rDNA DSB-induced transcriptional repression. **A** Schematic diagram of 4D-FastDIA quantitative proteomics analysis of EHMT2 substrates. **B** Principal Component Analysis (PCA) of samples from EHMT2 gRNA CTR and EHMT2 KO2 HeLa I-*Ppo*I cells. **C** Differentially expressed proteins in the EHMT2 gRNA CTR and EHMT2 KO2 HeLa I-*Ppo*I cells were classified for subcellular structural mapping. **D** Heatmaps of protein expression levels in HeLa I-*Ppo*I cells after rDNA DSBs showing differences between EHMT2 gRNA CTR and EHMT2 KO2 HeLa I-*Ppo*I cells. **E** Volcano plot showing the fold changes of all identified proteins and *P*-value in the cells between EHMT2 gRNA CTR and EHMT2 KO2 groups. **F** shRNA-based validation screen for EHMT2 targets involved in rDNA DSB-induced transcriptional suppression. HeLa I-*Ppo*I cells were infected with the indicated lenti-shRNA viruses. rDNA DSBs in cells were then induced with Shield-1 and 4-OHT and were subsequently subjected to EU incorporation assay. Relative nucleolar EU intensity was quantified from three independent experiments. **G** The knockdown efficiencies of the targeted shRNAs were validated by RT-qPCR. Data were from three independent experiments. Bars represent mean ± SEM; ns not significant; ^**^P < 0.01; ^****^P < 0.0001.

In addition to its catalytic role in methylating histone tails, EHMT2 coordinates chromatin-associated processes by recognizing histone methylation and recruiting specific protein complexes [20]. To delineate how EHMT2 mediates rDNA DSB-induced transcriptional silencing and rDNA DSB repair, we performed a global proteomic profiling of EHMT2 targets in cells following I-*Ppo*I induction. Briefly, EHMT2-KO HeLa I-*Ppo*I cells were subjected to rDNA DSB induction for 5 h, lysed and processed for proteomic analysis (Fig. 5A). Principal component analysis (PCA) revealed the characteristics of the samples (Fig. 5B). Bioinformatics analysis revealed a set of significantly dysregulated proteins in EHMT2-KO cells than in control cells following rDNA DSBs, which represented potential EHMT2 targets (Fig. 5C–E, Supplementary Fig. S4A, B, and Supplementary Table 7). Moreover, integrated functional enrichment analysis identified significantly associations between the differentially expressed proteins and key biological processes (Supplementary Fig. S4C–E). To identify novel EHMT2-dependent regulators of rDNA DSB-induced transcriptional silencing, we prioritized five candidates from the significantly downregulated proteins that could possibly be involved in DDR or transcriptional regulation using a short hairpin RNA (shRNA)-based validation screen to assess their potential as novel regulators of transcriptional suppression at rDNA DSBs (Fig. 5E). Intriguingly, we found that shRNA-mediated metallo-β-lactamase domain-containing protein 2 (MBLAC2) deficiency led to the significantly persistent nucleolar transcription after rDNA DSBs (Fig. 5F, G), implicating MBLAC2 as a novel regulator in the promotion of DSB-dependent transcriptional silencing in rDNA arrays.

### MBLAC2 promotes rDNA DSB-induced transcriptional suppression

**Fig. 6:**
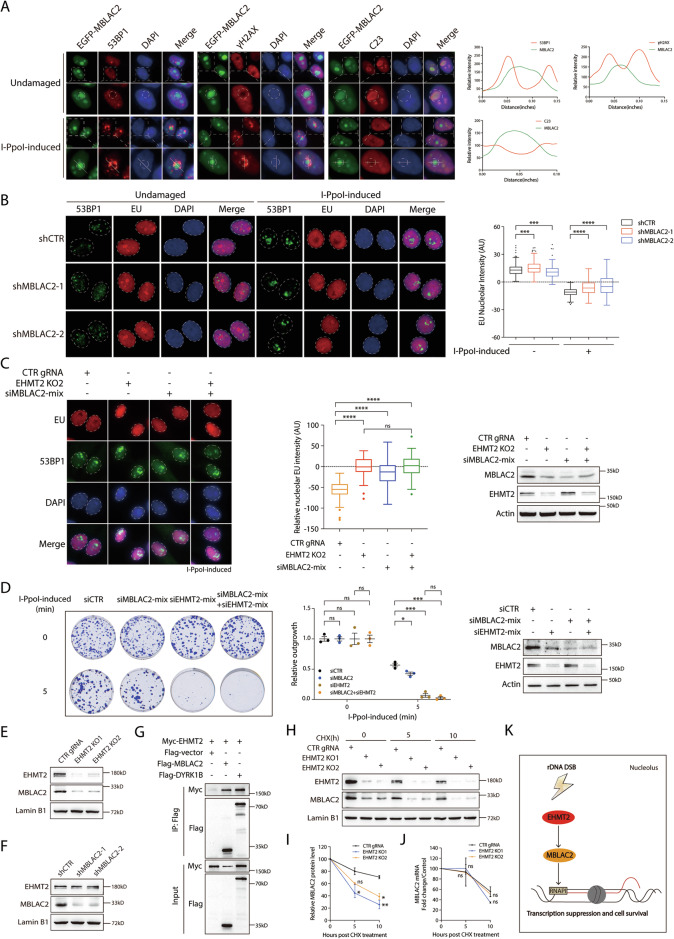
MBLAC2 promotes transcriptional silencing at rDNA DSBs. **A** The cellular localization of MBLAC2 in HeLa I-*Ppo*I cells following rDNA DSBs induction. HeLa I-*Ppo*I cells were transfected with EGFP-MBLAC2 and were treated with Shield-1 and 4-OHT for 4 h. Fixed cells were labeled with anti-53BP1, anti-γH2AX or anti-C23 antibodies. Nuclei were counterstained with DAPI. Enlarged images show the details of the indicated proteins. Quantification of relative signal intensities of EGFP-EHMT2, 53BP1, γH2AX and C23 was performed by ImageJ. The white lines in the enlargements of the representative images indicate the lines for analysis. The edge of the indicated nucleolar caps was labeled with dotted circle. **B** Analysis of nucleolar transcription activity by EU incorporation assay in MBLAC2-inactivated HeLa I-*Ppo*I cells following rDNA DSBs induction. Cells transduced with control (shCTR) or two independent MBLAC2-targeted shRNAs were induced for rDNA DSBs for 4 h. EU nucleolar intensity was subsequently determined by EU incorporation assay. At least 200 cells exhibiting well-circumscribed nucleoli were quantitatively assessed across two independent experiments. Quantification of relative EU nucleolar intensity is shown in Tukey boxplots. **C** Nucleolar EU intensities were analyzed in HeLa I-*Ppo*I cells treated with the EHMT2 gRNA or MBLAC2 siRNA after I-*Ppo*I induction. Relative nucleolar EU intensity was quantified from at least two independent experiments. Immunoblot of MBLAC2 and EHMT2 in the HeLa I-*Ppo*I cells induced with the indicated gRNA or siRNA. **D** Colony survival of HeLa I-*Ppo*I cells transfected with CTR siRNA and siRNA targeting MBLAC2 or EHMT2 following I-*Ppo*I induction, respectively. Cells were induced for rDNA DSBs for 5 min. After washing with PBS twice, cells were allowed to grow for two weeks before harvest and Coomassie blue staining. The relative outgrowth of the colonies between groups were quantified and plotted. The protein expression of MBLAC2 and EHMT2 in HeLa I-*Ppo*I cells transfected with the indicated siRNAs were examined by immunoblot. **E** Immunoblot of MBLAC2 and EHMT2 in the HeLa I-*Ppo*I cells transduced with control gRNA (CTR gRNA) and two EHMT2 gRNAs (EHMT2 KO1 and EHMT2 KO2). **F** shRNA-mediated MBLAC2 knockdown efficiency was measured by Western blotting. **G** The EHMT2-MBLAC2 interaction was confirmed by Co-immunoprecipitation (Co-IP). Flag-DYRK1B was used as the positive control. **H** HeLa I-*Ppo*I cells transduced with control gRNA (CTR gRNA) and two EHMT2 gRNAs (EHMT2 KO1 and EHMT2 KO2) were treated with cycloheximide for 0, 5, and 10 h. The protein expression of MBLAC2 and EHMT2 in HeLa I-*Ppo*I cells was examined by immunoblot. **I** The relative MBLAC2 protein level in HeLa I-PpoI cells treated with cycloheximide as depicted in (**A**) was measured. Data were derived from three independent experiments. **J** HeLa I-PpoI cells were transduced with control gRNA (CTR gRNA) and two EHMT2 gRNAs (EHMT2 KO1 and EHMT2 KO2). Fold change of MBLAC2 mRNA in I-*Ppo*I cells treated with cycloheximide was determined by RT-qPCR. Quantification of MBLAC2 mRNA fold change was from three independent experiments. **K** Proposed working model of EHMT2-MBLAC2 axis in promoting rDNA DSB-induced transcriptional suppression. Bars represent mean ± SEM; ns not significant; ^*^P < 0.05; ^**^P < 0.01; ^***^P < 0.001; ^****^P < 0.0001.

MBLAC2 is a poorly characterized protein reported to possess acyl-CoA hydrolase activity, and its potential role in the DNA damage response remains elusive [29]. As our shRNA-based validation screen revealed that MBLAC2 participates in the rDNA DSB response, we examined whether it accumulates at the nucleolar periphery after I-*Ppo*I induction. Immunofluorescence analysis revealed that MBLAC2 constitutively localized to the nucleolus. In contrast with the DSB markers γH2AX and 53BP1 or the nucleolar protein C23, its distribution was unaffected by rDNA break induction (Fig. 6A). shRNA-mediated MBLAC2 knockdown (KD)resulted in a defect in rDNA DSB-induced transcriptional shutdown (Fig. 6B), further demonstrating that MBLAC2 promoted RNAPI transcriptional suppression in the presence of rDNA DSBs. Moreover, the absence of an additive effect on nucleolar EU incorporation in the EHMT2-MBLAC2 combinatorial depletion compared to that in EHMT2 monogenic KO indicated that MBLAC2 promoted rDNA DSB-induced transcriptional repression linearly within the same EHMT2-dependent pathway (Fig. 6C). To evaluate whether MBLAC2 is indispensable for cellular tolerance to rDNA DSBs, we performed clonogenic survival assays. I-*Ppo*I induction appreciably decreased in the number of MBLAC2-deficient colonies compared to that of control cells, along with a lack of synthetic enhancement in EHMT2-MBLAC2 double depletion, suggesting MBLAC2 as a core component of the EHMT2-mediated pathway essential for cell survival (Fig. 6D). Notably, in agreement with the proteomic profile, genetic ablation of EHMT2 and siRNA-mediated EHMT2 KD drastically reduced MBLAC2 expression (Fig. 6E and Supplementary Fig. S5A), though not vice versa (Fig. 6F), suggesting that MBLAC2 could serve as a downstream effector in the EHMT2-mediated transcriptional silencing pathway. Indeed, we found that MBLAC2 immunoprecipitated with EHMT2, indicating the EHMT2-MBLAC2 complex formation, and supporting MBLAC2 protein stability (Fig. 6G). To investigate the functional significance of the EHMT2-MBLAC2 complex in maintaining MBLAC2 stability, we assessed MBLAC2 protein turnover using a cycloheximide chase assay. In agreement with the crucial role of EHMT2 binding in conferring MBLAC2 protein stability, loss of EHMT2 resulted in significantly higher turnover rates compared to that in control cells (Fig. 6H, I), without affecting its mRNA levels (Fig. 6J). These findings were corroborated in EHMT2-knockdown cells (Supplementary Fig. S5B, C). Furthermore, we found that EHMT2 and MBLAC2 depletion had no significant effect on cell cycle stages, indicating that EHMT2 and MBLAC2 facilitate rDNA DSB responses in a cell cycle-independent manner (Supplementary Fig. S6A, B).

Taken together, these results suggested that EHMT2 maintains nucleolar MBLAC2 protein stability to promote rDNA DSB-induced transcriptional silencing and cell viability following rDNA DSBs (Fig. 6K).

### The EHMT2-MBLAC2 signaling axis correlates with colorectal tumorigenesis

**Fig. 7:**
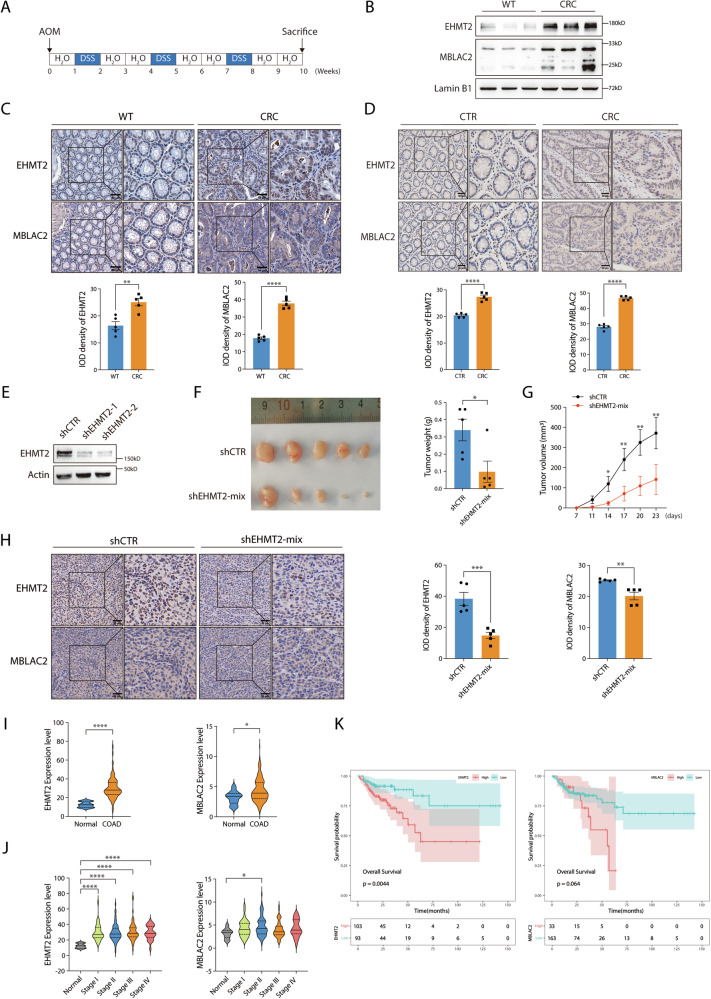
EHMT2-MBLAC2 signaling axis correlates with tumorigenesis. **A** Schematic illustration depicts the establishment of azoxymethane/dextran sulfate sodium (AOM/DSS)-induced colorectal cancer (CRC) mice model. **B** Immunoblot of EHMT2 and MBLAC2 in colon specimen derived from wild-type (WT) and colorectal cancer mice. **C** Representative immunohistochemistry (IHC) images stained with EHMT2 and MBLAC2 in colon or colon tumors from WT and CRC tissues, respectively. Scale bar = 50 μm. The enlarged pictures show the details of EHMT2 and MBLAC2 staining. Integrated optical density (IOD) of EHMT2 and MBLAC2 were quantified based on five IHC images derived from three independent experiments. **D** Representative images of IHC stained with EHMT2 and MBLAC2 in colon tumors and adjacent normal tissues from human patient specimens. Scale bar = 100 μm. The enlarged pictures show the details of EHMT2 and MBLAC2 staining. The IOD of EHMT2 and MBLAC2 from representative IHC images were quantified as described in (**C**). **E** Immunoblot of EHMT2 in HCT 116 cells transduced with control (shCTR) and EHMT2-targeting shRNAs (shEHMT2-1 and shEHMT2-2). Actin was used as loading control. **F** Representative images of excised tumors from nude mice subcutaneously injected with either control (shCTR) or shRNA-mix (shEHMT2-1 and shEHMT2-2) transduced HCT 116 cells. Tumor weight was quantified. **G** Tumor growth curve displaying the dynamics of tumor volume as assessed by caliper measurement. **H** Representative IHC images of EHMT2- and MBLAC2-stained tumor sections derived from (**F**). Scale bar = 50 μm. The enlarged pictures show the details of staining. Quantification of IOD of EHMT2 and MBLAC2 from five representative IHC images. **I** Analysis of EHMT2 and MBLAC2 expression levels using RT-COAD datasets. The expression of EHMT2 (left) and MBLAC2 (right) between normal and colon adenocarcinoma were quantified. **J** The correlation between EHMT2 (left) or MBLAC2 (right) expression and different tumor stages in RT-COAD patients was analyzed. **K** Survival analysis of the RT-COAD data on patients with high or low expression levels of EHMT2 (left) and MBLAC2 (right) as described in (**J**). Bars represent mean ± SEM; ^*^P < 0.05; ^**^P < 0.01; ^***^P < 0.001; ^****^P < 0.0001.

Dysregulation of rDNA repair and copy number is linked to human malignancies due to its role in undermining genomic stability [5, 9]. Colorectal cancer (CRC) exhibits a hallmark dependency on hyperactive ribosome biogenesis, creating a unique therapeutic vulnerability [30]. This intrinsic vulnerability is clinically exploited by most CRC chemotherapeutic agents such as Oxaliplatin, which functions by inducing ribosomal stress [31, 32]. Emerging evidence has implicated the role of EHMT2 in CRC [33–35]. It was identified as an immunotherapeutic target in microsatellite stable (MSS) CRC through an epigenetic screen and as a target of human gut-microbiome-derived propionate-induced apoptosis in colon cancer [34, 35]. However, its precise roles and underlying mechanisms remain incompletely understood. To consolidate the role of EHMT2-MBLAC2 axis in rDNA DSB-induced transcriptional silencing in colon cancer cells, we performed the EU incorporation assays in either EHMT2- or MBLAC2-knockdown HCT 116 I-*Ppo*I cells. Consistently, we found that EHMT2 or MBLAC2 depletion led to the compromised rDNA DSB-induced transcriptional silencing (Supplementary Fig. S7A–D). Moreover, cell viability was measured by CCK-8 assays in a panel of colon cancer cells (HCT-8, HCT-15, and HCT 116) upon Oxaliplatin treatment. EHMT2 or MBLAC2 deficiency rendered colon cancer cells more susceptible to Oxaliplatin, suggesting EHMT2 and MBLAC2 promote cell viability of colon cancer cells under ribosomal stress (Supplementary Fig. S8A–D). To further investigate the role of the EHMT2–MBLAC2 axis in CRC tumorigenesis, we evaluated the expression of both EHMT2 and MBLAC2 in CRC tissues. In the colon tissues of a mouse model of azoxymethane/dextran sulfate sodium (AOM/DSS)-induced CRC, we found the upregulated expression of both EHMT2 and MBLAC2 than in their wild-type (WT) controls (Fig. 7A, B), which was consistent with the immunohistochemistry results derived from the same tumor tissues (Fig. 7C). Furthermore, we observed increased expression of ribosome biogenesis markers UBF and RPL5 in mouse CRC tissues (Supplementary Fig. S9A), suggesting the enhanced ribosome biogenesis during colorectal tumorigenesis. Consistent with this, immunohistochemical staining confirmed the concurrent upregulation of EHMT2 and MBLAC2 in human CRC tissues (Fig. 7D), indicating that the aberrant expression of EHMT2 and MBLAC2 in colorectal tumors could be correlated with colorectal tumorigenesis.

To determine whether the EHMT2-MBLAC2 signaling axis plays an oncogenic role in tumorigenesis, we used shRNA-mediated EHMT2-deficient colorectal HCT 116 cells in a xenograft assay (Fig. 7E). EHMT2 knockdown significantly suppressed tumor growth, as evidenced by the reduced tumor weight and volume in the HCT 116 xenograft models (Fig. 7F, G), suggesting that EHMT2 promotes colorectal tumorigenesis. As expected, substantially reduced MBLAC2 expression was observed in EHMT2-deficient HCT 116 xenografts using immunohistochemistry, validating the essential role of EHMT2 in maintaining MBLAC2 stability in a tumorigenic context (Fig. 7H). Furthermore, the reduced expression of UBF and RPL5 in EHMT2-deficient HCT 116 xenografts suggested that EHMT2 is required to sustain ribosome biogenesis during colorectal tumorigenesis (Supplementary Fig. S9B). Moreover, analysis of transcriptomic data from a radiotherapy-treated colon adenocarcinoma (RT-COAD) cohort in TCGA revealed that both EHMT2 and MBLAC2 were significantly overexpressed in tumor tissues relative to adjacent non-tumor tissues (Fig. 7I). Interestingly, we observed a correlation between the expression of EHMT2-MBLAC2 and CRC disease stage in the RT-COAD dataset (Fig. 7J), indicating the potential role of EHMT2-MBLAC2 signaling axis in CRC progression. To assess the prognostic significance of the EHMT2–MBLAC2 axis in CRC treated with radiotherapy, we analyzed RT-COAD dataset and revealed that high expression of both EHMT2 and MBLAC2 displayed poor overall survival in the COAD patients treated with radiotherapy (Fig. 7K). Moreover, the correlation of RT-COAD dataset revealed that both EHMT2 and MBLAC2 were positively correlated with known regulators of rDNA DSB-dependent silencing, such as ATR, Treacle, TopBP1 and HUSH subunits (Supplementary Fig. S10A, B). Collectively, our findings indicated a critical role of the EHMT2–MBLAC2 signaling axis in promoting colorectal tumorigenesis.

## Discussion

**Fig. 8:**
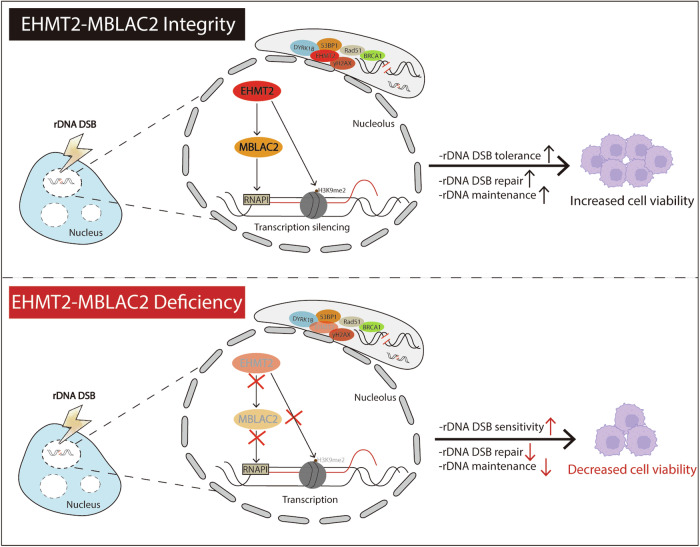
Schematic model on EHMT2-MBLAC2 signaling axis in rDNA DSB-induced transcriptional silencing and tumorigenesis. EHMT2 suppresses rDNA transcription by either targeting MBLAC2 or regulating deposition of H3K9me2 at chromatin upon rDNA DSBs induction. The EHMT2-MBLAC2 signaling axis promotes rDNA DSB repair, maintains rDNA stability and cell DSB tolerance in response to rDNA breaks. Additionally, DYRK1B facilitates EHMT2 accumulation at nucleolar caps. Inhibition of EHMT2-MBLAC2 axis compromised nucleolar responses, especially rDNA DSB-induced transcriptional silencing and cell viability, shedding light on the importance of rDNA instability in tumorigenesis.

In this study, we found that the EHMT2-MBLAC2 signaling axis suppresses rDNA transcription in response to rDNA DSBs. Following I-*Ppo*I-induced rDNA DSBs, EHMT2 localizes to the nucleolar periphery to form caps and is essential for RNAPI-dependent transcriptional silencing (Fig. 1B–G, Fig. 2A–E and Supplementary Fig. S1D). EHMT2-mediated nucleolar transcriptional silencing occurs independently of nucleolar segregation (Fig. 3A–F). Additionally, DYRK1B phosphorylates EHMT2 at T555, which drives EHMT2 accumulation in the rDNA DSB-induced nucleolar caps (Supplementary Fig. S2A–C). We also revealed that EHMT2 fine-tunes rDNA DSB repair to maintain rDNA stability (Fig. 4A and Supplementary Fig. S3A–D). More importantly, EHMT2 deficiency led to increased chromosomal instability and cellular hypersensitivity to nucleolar damage (Fig. 4B–H and Supplementary Fig. S3E). By conducting a global proteomic analysis of EHMT2 in I-*Ppo*I-induced cells, we further identified MBLAC2 as a key downstream effector that suppresses RNAPI transcription upon rDNA damage (Fig. 5A–G). EHMT2 stabilized MBLAC2 and loss of MBLAC2 phenocopied EHMT2 deficiency in nucleolar DDRs (Fig. 6A-I and Supplementary Fig. S5A, B). Furthermore, we found that the EHMT2-MBLAC2 signaling axis promotes colorectal tumorigenesis (Fig. 7A–H). Together, our findings highlighted the EHMT2-MBLAC2 signaling axis as a key branch of the mammalian nucleolar DDRs network (Fig. 8).

Although rDNA DSB mobilization to the nucleolar periphery involves DSB repair, the underlying mechanism of the latter in the nucleolar caps remains unclear. DSB mobilization within the nucleolar caps is predominantly repaired by HR [12, 13], or DNA-PK-dependent NHEJ repair [7]. Consistent with previous findings, we observed the rDNA DSB-induced accumulation of BRCA1 and 53BP1 at the periphery of the nucleoli, implying that rDNA breaks within the nucleolar caps could be repaired by both HR and NHEJ [12]. We found robust accumulation of HR core factors (Rad51 and BRCA1) and mild recruitment of NHEJ factors (Ku80 and DNA-PKcs) at the periphery of the nucleoli, inconsistent with a previous report, and no evident enrichment of NHEJ factors at nucleolar caps [12]. Interestingly, EHMT2 deficiency specifically impaired the formation of NHEJ-related nucleolar caps (Supplementary Fig. S3A–D), suggesting that EHMT2 might promote the rDNA damage within the nucleolar caps by NHEJ. Additionally, EHMT2 mediates cell cycle-independent repair of nucleolar caps (Supplementary Fig. S6A). While the mechanisms governing repair pathway choice in nucleolar caps require further investigation, our findings nevertheless demonstrate that EHMT2 is required for the faithful repair of rDNA DSBs, as its deficiency leads to persistent rDNA damage and the loss of rDNA repeat.

rDNA DSB-induced transcriptional repression has been associated with nucleolar segregation evidenced by the formation of nucleolar caps [7, 25]. However, the mechanism underlying rDNA DSB-induced transcriptional silencing may vary among the key components in RNAPI-mediated transcriptional arrest in response to rDNA DSBs. Although ATM-dependent rDNA damage signaling triggers transcriptional silencing alongside nucleolar reorganization [7, 12], our recent work revealed that DYRK1B loss led to a defect in rDNA transcriptional silencing without affecting nucleolar reorganization to uncouple the distinct mechanism of DSB-induced rDNA transcriptional repression from the established ATM-dependent pathway [18]. Similarly, our results indicated that EHMT2 facilitated rDNA DSB-induced transcriptional suppression, independent of nucleolar chromatin reorganization (Fig. 3A–F). However, further investigations would be required to elucidate the molecular pathways of these distinct nucleolar DDRs.

Global proteomic analysis of EHMT2 revealed MBLAC2 as a downstream effector that promotes rDNA DSB-induced transcriptional suppression (Fig. 5E–G). Although we established that MBLAC2 localizes to the nucleolus and its loss sustains rRNA incorporation and confers hypersensitivity to rDNA DSBs (Fig. 6A–D), the precise mechanism by which it regulates RNA Polymerase I activity requires further investigation. Moreover, EHMT2 stabilized and interacted with MBLAC2 to silence RNAPI transcription upon rDNA DSBs (Fig. 6E–I), suggesting that the EHMT2-MBLAC2 signaling axis might function as a novel branch of nucleolar DDRs that modulates rDNA transcription at DSBs. Furthermore, EHMT2-MBLAC2 promoted DSB-induced rDNA transcription suppression independent of the cell cycle stage (Supplementary Fig. S6A, B).

Genome instability is regarded as a hallmark of cancer and rDNA arrays are exceedingly susceptible to DSBs, making it a mutational hotspot in cancer [9]. Interestingly, EHMT2 or MBLAC2 deficiency sensitizes colorectal cancer cells to nucleolar transcription inhibitor Oxaliplatin (Supplementary Fig. S8A–D), indicating a critical role for rDNA transcription in sustaining colorectal cancer cell survival. To investigate the potential role of the EHMT2-MBLAC2 axis in colorectal tumorigenesis, we found consistent overexpression of EHMT2-MBLAC2 in both AOM/DSS-induced mouse colorectal tumors and clinical specimens from patients with CRC (Fig. 7A–D). EHMT2 deficiency led to the inhibition of HCT 116 cell xenograft growth (Fig. 7E–G) and the downregulation of MBLAC2 expression (Fig. 7H). Interestingly, we found the decreased expression of UBF and RPL5 in the EHMT2-depleted HCT 116 cell xenografts, suggesting EHMT2 is required for ribosome biogenesis during CRC tumorigenesis (Supplementary Fig. S9A, B). Analysis of RT-COAD data further established the high expression of EHMT2 and MBLAC2 as a predictor of poor prognosis in COAD patients treated with radiotherapy (Fig. 7K). Furthermore, analysis of TCGA data from the RT-COAD cohort demonstrated that the expression levels of both EHMT2 and MBLAC2 exhibited significant positive correlations with established rDNA DSB-dependent transcriptional silencing regulators, such as ATR, Treacle, TopBP1, and HUSH subunits, corroborating the role of EHMT2 and MBLAC2 in the regulatory network of rDNA DSB-induced transcriptional silencing (Supplementary Fig. S10A, B). However, the current results did not directly address the role of EHMT2-MBLAC2 axis-mediated ribosomal DDRs in vivo. Further studies would be required to decipher how the EHMT2-MBLAC2 axis coordinates rDNA transcription inhibition with rDNA DSB repair and how it translates to colorectal tumorigenesis, shedding light on the role of nucleolar DDR factors in human diseases, particularly in cancer.

In conclusion, we identified EHMT2 and its downstream effector MBLAC2 as the novel branch of nucleolar DDRs that promotes rDNA DSB-induced transcription silencing. Additionally, the EHMT2-MBLAC2 signaling axis is essential for rDNA DSB repair, and the maintenance of rDNA integrity and cell viability following I-*Ppo*I-induced rDNA DSBs. It is implicated in colorectal tumorigenesis and its high expression predicts poor survival in radiotherapy-treated colon adenocarcinoma patients.

## Materials and methods

### Cell culture

HeLa, U2OS, GES1 and HEK293T cells were obtained from ATCC and SW480 cells were obtained from Servicebio. These cells were cultured in Dulbecco’s Modified Eagle Medium (DMEM) (Gibco) media supplemented with 10% fetal bovine serum (FBS) (Gibco) and 1% penicillin/streptomycin (Gibco). The colon cancer cell lines HCT 116, HCT-15, and HCT-8 (obtained from COBIOER) were maintained in Roswell Park Memorial Institute 1640 Medium (RPMI-1640) (Gibco) containing 10% FBS (Gibco) and 1% penicillin/streptomycin (Gibco). Cells were incubated at 37 °C under a standard humidified atmosphere of 5% CO₂.

### Plasmids

Plasmids used in this study are listed in the Supplementary Table 1.

### Antibodies

Antibodies used for immunofluorescence (IF) staining: anti-γH2AX (EMD Millipore, #JBW301); Anti-53BP1 (Novusbio, #NB100-304); Fibrillarin(CST, 2639T); anti-Ki-67 (Abcam, #ab15580); anti-C23 (Nucleolin; CST, #14574); anti-UBF (F-9) (Santa Cruz, #sc-13125); anti-BRCA1 (Santa Cruz, #sc-6954); Anti-Rad51 (Santa Cruz, # sc-398587); anti-Myc (Abclonal, #AE070); anti-GFP (Proteintech, #66002-1); Alexa Fluor 594 AffiniPure Goat Anti-Mouse IgG (H + L) (Jackson ImmunoResearch, #115-585-166); Alexa Fluor 488 AffiniPure Goat Anti-Rabbit IgG (H + L) (Jackson ImmunoResearch, #111-545-003); Alexa Fluor 488 AffiniPure Goat Anti-Mouse IgG (H + L) (Jackson ImmunoResearch, #115-545-003); Alexa Fluor™ Plus 405 Anti-Rabbit (Invitrogen, #A48254); Alexa Fluor™ Plus 405 Anti-Mouse (Invitrogen, #A48255).

Antibodies used for Western blotting experiments: EHMT2(CST, #3306S); EHMT2(Abclonal, #A19288); EHMT2(Proteintech, #66689-1-Ig); EHMT2(Selleck, #F0379); MBLAC2(Santa Cruz, #SC-398153); MBLAC2(Abmart, #PS13383); H3K9me2(CST, #4658S); Lamin B1(Sharebio, #SB-AB0054); anti-Flag (Abcam, #ab20560); anti-Myc (Abclonal, #AE070); anti-β-Actin (Abclonal, #AC004); anti-γH2AX (EMD Millipore, #JBW301); Anti-53BP1 (Novusbio, #NB100-304). Peroxidase AffiniPure Rabbit Anti-mouse IgG+IgM (H + L) (Jackson ImmunoResearch, #315-035-048); Peroxidase AffiniPure Goat Anti-Rabbit IgG (H + L) (Jackson ImmunoResearch, #111-035-144).

Antibodies used for IHC: EHMT2(Abclonal, #A19288); MBLAC2(Santa Cruz, #SC-398153); MBLAC2(Abmart, #PS13383); anti-UBF(Santa Cruz, sc-13125); RPL5(Abclonal, #A1977).

### Inhibitors

ATM inhibitor (KU-55933, Selleckchem, #S1092, 10 μM); EHMT2 inhibitor (UNC0638, Selleckchem, #S8071, 10 μM); DMSO (Sigma-Aldrich, #D2650); Nucleolar transcription inhibitor (Oxaliplatin, MCE, #HY-17371).

### Lentivirus packaging and cell line generation

HeLa/U2OS /GES1 I-*Ppo*I cells: HEK293T cells were co-transfected with pLVX-PTuner-I-*Ppo*I, psPAX2 (Addgene, plasmid#12260) and pMD2.G (Addgene, plasmid#12259) through polyethylenimine (PEI) (Polysciences, #23966) to generate lentivirus. The medium containing pLVX-PTuner-I-*Ppo*I lentivirus was then harvested at 48 h post-transfection and filtered with Syringe Filter using filter with 0.45 μm membrane (PALL Life Sciences). Cells were infected with lentivirus in the presence of 8 μg/ml polybrene (Solarbio, #H8761) for 24 h. Cells were then selected in the culturing media with 1 μg/ml puromycin (Solarbio, #P8230) to establish stable cell line.

CRISPR/Cas9-engineered knockout (KO) and shRNA-mediated knockdown (KD) cells: non-targeting control gRNA and single guide RNAs (sgRNAs) targeting sequences were cloned into LentiCRISPR v2 vector (Addgene, plasmid#52961) following the cloning protocol from Feng Zhang’s Lab. Lentivirus was packaged as described above. Cells were incubated with the media containing lentivirus supplemented with 8 μg/ml polybrene. After puromycin selection, polyclonal stable cells were generated, with subsequent validation of knockout efficiency by Western blotting. Sequences of gRNAs and shRNAs are listed in Supplementary Tables 2, 3.

### I-*Ppo*I induction

I-*Ppo*I cells were treated with the medium supplemented with 1 μM Shield-1 (Selleck, #S3469) and 2 μM 4-OHT (Selleck, #S8956) for the indicated period of time to activate I-*Ppo*I endonuclease to induce nucleolar rDNA DSBs.

### Small interfering RNA (siRNA)-mediated knockdown

Cells were transfected with non-targeting control or gene-specific siRNAs (Genepharma) using RFect siRNA Transfection Reagent (Biodai, #11012) for 48 h before subjecting to the following experiments. Sequences of siRNAs are listed in Supplementary Table 4.

### EU incorporation assay

Nucleolar transcription activity was determined by BeyoClick™ EU RNA Synthesis Kit with Alexa Fluor 594 (Beyotime, #R0309S) following manufacturer’s guidelines. Briefly, pre-treated cells grown on coverslips were incubated with medium containing recommended 5-ethynyl uridine (EU) concentration for 1 h followed by subsequent fixation, permeabilization, and EU detection. Cells were immunostained with DSB markers γH2AX and 53BP1 or nucleolar markers Fibrillarin (FBL) and UBF antibodies. Cells were washed three times with PBS for 5 min and mounted with Antifade Mounting Medium (Servicebio, #G1401). After mounting, cells were subjected to image acquisition using the wide-field microscope (Olympus BX53). Quantitative analysis of EU nucleolar intensity was performed by ImageJ software (NIH) using the regions-of-interest (ROI) manager. Given the precise colocalization of DSB markers γH2AX and 53BP1 or nucleolar markers Fibrillarin (FBL) and UBF at the nucleolar periphery, we used these nucleolar or DSB marker-positive nucleolar caps as spatial landmarks to define nucleolar boundaries following I-*Ppo*I-induced rDNA DSBs. The fluorescent intensity profiles of EU were quantified by calculating the ratio of EU reads within nucleolar caps-labeled nucleoli to that in the adjacent peri-nucleolar regions within the same nuclei. Only nucleoli that were large and exhibited distinct margins were included in the analysis. At least 150 cells exhibiting well-circumscribed nucleoli were quantitatively assessed across minimally two independent experiments.

### Western blotting and co-immunoprecipitation (co-IP)

Harvested cells were washed with PBS and lysed with ice-cold NETN lysis buffer [20 mM Tris·HCl (pH 8.0), 100 mM NaCl, 0.5% Nonidet P40, and 1 mM EDTA] supplemented with Benzonase nuclease (Sigma, #E1014) and proteasome inhibitor (APE × BIO, #K1007) for 30 min on ice. Whole cell lysates were subsequently resolved in SDS-PAGE gels and electrotransferred onto PVDF membranes. Membranes were blocked in 5% non-fat milk in Tris-buffered saline+Tween 20 (TBST) for 1 h at room temperature and then were incubated with primary antibodies diluted in 3% bovine serum albumin (BSA) /TBST at 4 °C overnight. After TBST washes, membranes were incubated with HRP-conjugated secondary antibodies for 1 h at room temperature. Proteins were detected by chemiluminescence solutions (Millipore) using Tanon 5200 Multi automatic chemiluminescence imaging system (Tanon). For co-immunoprecipitation, cells were transiently transfected with the indicated plasmids using PEI and cells were harvested at 48 h post-transfection. Whole cell lysates were centrifuged at 13,000 rpm for 15 min at 4 °C, the clarified supernatants were then incubated with anti-Flag Magnetic Beads with continuous gentle rotation (MCE, #HY-K0207) at 4 °C for 4 h. The bead-bounded protein complexes were subsequently washed three times with ice-cold NETN buffer prior to immunoblotting with the anti-Flag and anti-Myc antibodies.

### Immunofluorescence microscopy

For immunofluorescence microscopy experiments, cells seeded onto coverslips in 6-well plate were fixed with 4% paraformaldehyde (PFA) for 30 min and permeabilized with 0.5% Triton X-100 for 1 min at room temperature. After washing twice with 1× PBS for 5 min, cells were then incubated with primary antibodies diluted in 3% BSA/TBST for 1 h at room temperature. Appropriately diluted secondary antibodies (Alexa Fluor 488 and Alexa Fluor 594 antibodies) were added onto the coverslips for another 1 h at room temperature followed by DAPI (MCE, #HY-D0814) staining. Finally, coverslips were washed 3 times with 1× PBS for 5 min and mounted with Antifade Mounting Medium (Servicebio, #G1401). Coverslips carrying fixed cells were then imaged on a wide-field fluorescence microscope (Olympus BX53) for image acquisition.

### Neutral comet assay

Following treatment, cells were resuspended in ice-cold DPBS at the density of 5 × 10^5^ cells/ml and gently mixed with pre-warmed 37 °C molten LMAgarose (R&D, #4250-050-02) at a 1:10 (v/v) ratio. The suspension was immediately pipetted onto preheated 37 °C comet slides (R&D, #4250-200-03) and evenly distributed. The slides were subsequently solidified in darkness at 4 °C for 30 min and were then immersed in chilled CometAssay Lysis Solution (R&D, #4250-050-01) at 4 °C for 1 h. Slides were washed with 1× TBE (Tris-Borate-EDTA) buffer twice before electrophoresis in freshly prepared TBE at 4 °C under light-proof conditions. After electrophoresis, slides were incubated in DNA precipitation solution for 30 min at room temperature followed by fixation in 70% ethanol for 5 min. After air-drying, slides were counterstained with 1 μg/ml propidium iodide (Solarbio, #C0080) for 30 min at room temperature in darkness. Slides were rinsed in distilled water and were mounted with antifade medium. Images were acquired by an Olympus BX53 microscope and DNA damage quantification was analyzed with OpenComet plugin by ImageJ (NIH) software.

### RNA isolation and RT-qPCR

RNA extraction was performed with Super FastPure Cell RNA isolation kit (Vazyme, #RC102-01) following the manufacturer’s instructions. 1 μg RNA was processed for reverse transcription using 4× Hifair® AdvanceFast One-Step RT SuperMix (Yeasen, #11151). The quantitative real-time PCR was processed with the diluted cDNA, the indicated primers and 2× SYBR Green qPCR Master Mix (Selleck, #B21202) on LightCycler 480II RT-PCR System (Roche Applied Science). Data were quantified by 2^−ΔΔCT^ method with GAPDH used as the internal reference gene. Primers used for RT-qPCR are listed in Supplementary Table 5.

### 4D-FastDIA proteomic analysis

HeLa I-*Ppo*I cells transduced with CTR gRNA or EHMT2 KO2 were induced for rDNA DSBs and cells were harvested at 5 h after I-*Ppo*I induction. After three washes with ice-cold PBS, cell pellet was snap-frozen in liquid nitrogen using pre-chilled cryovials and subsequently stored at −80 °C until further processing. The 4D-FastDIA proteomic analysis was performed by PTM-Biolabs (Hangzhou, China). Briefly, cell samples were then lysed using the lysis buffer (8 M urea, 1% protease inhibitor cocktail). After determining the concentrations of the extracted protein, the samples were subjected to trypsin digestion before high-resolution liquid chromatography–mass spectrometry (LC–MS/MS) analysis. The DIA data were processed using DIA-NN search engine (v.1.8) and subjected to mass spectrometry data analysis. All the peptides with an adjusted *P*-value < 0.05 were defined as upregulation or downregulation, respectively. In differential analysis, when the *P*-value < 0.05, a fold change greater than 1.5 was considered as the threshold for significant upregulation, and less than 1/1.5 as the threshold for significant downregulation.

### Genomic DNA extraction and real-time qPCR

Genomic DNA extraction was performed with TIANamp Genomic DNA Kit (TIANGEN, DP304-02) following the manufacturer’s recommendation. Genomic DNA was subjected to real time PCR and analyzed as described above. Primers used for genomic DNA qPCR are listed in Supplementary Table 6.

### Cell cycle analysis

Harvested cells were centrifuged at 1000 rpm/min for 5 min at room temperature and washed twice with PBS. The cell pellet was resuspended in ice-cold PBS and then fixed by dropwise addition of ice-cold 70% ethanol over a low-speed vortex. After incubation at −20 °C overnight, fixed cells were washed once with PBS and treated with 0.8% sodium citrate solution containing 200 μg/mL RNase A for 30 min at room temperature. Subsequently, fixed cells were stained with 50 μg/mL propidium iodide in darkness. Single-cell suspensions were obtained by filtration through a 40 μm cell strainer prior to flow cytometric analysis using Gallios flow cytometer (Beckman coulter).

### Micronuclei (MNs) measurement

Following treatment, cells were fixed in 4% PFA for 15 min at room temperature and subsequently permeabilized with 0.5% Triton X-100. Cells were counterstained with DAPI and imaged by fluorescence microscopy to quantify micronuclei. A minimum of 600 cells were scored from three independent biological replicates.

### Clonogenic survival assay

HeLa I-*Ppo*I cells were seeded on 60 mm dishes. At 24 h after seeding, cells were treated with Shield-1 (Selleck, #S3469, 1 μM) and 4-OHT (Selleck, #S8956, 2 μM) for 5, 10 or 20 min to induce rDNA DSBs. Following washes twice with PBS, cells were cultured in medium supplemented with 10% FBS for two weeks before fixation. Colonies were stained with Coomassie Blue Staining Solution and manually counted.

### Cycloheximide chase experiment

Cells plated in 60-mm dishes were treated with 50 μg/mL cycloheximide (MCE, #HY-12320). At the indicated time points, cells were harvested and subjected to Western blot analysis. Protein band intensities were quantified using ImageJ software (version 1.54p) and normalized to the untreated control.

### Cell counting kit-8 (CCK-8) assay

To assess the effect of EHMT2 and MBLAC2 knockdown on cellular sensitivity to Oxaliplatin, cell viability was evaluated using the Cell Counting Kit-8 (CCK-8) assay. HCT-8 and HCT-15 cells were transfected with siRNAs targeting EHMT2 or MBLAC2 to achieve protein knockdown. Stable knockdown in HCT 116 cells was achieved by transduction with lentiviral vectors expressing short hairpin RNAs (shRNAs). Following knockdown, cells were seeded into 96-well plates. After cell attachment, they were treated with the indicated concentrations of Oxaliplatin (MCE, #HY-17371) for 6 h. The medium was then replaced with fresh culture medium. Upon reaching ~80% confluence, cells were treated with CCK-8 reagent (APE X BIO, #K1018). Following a 4-h incubation, absorbance at 450 nm was measured with an Infinite E Plex reader (#M200pro) for viability determination.

### Subcutaneous xenograft assay

shRNA-mediated EHMT2-deficient HCT 116 cells were trypsinized, quantified and then resuspended in PBS at the concentration of 1 × 10^7^ cells/ml (two independent repeats). Sample sizes were determined empirically from previous experimental experience and were generally utilized in the field. Mice were randomly allocated into experimental groups. Then 100 μl resuspended cells were injected into right anterior armpit of 5-week-old male BALB/c nude mice (GemPharmatech). One week after injection, the length and width of tumor was measured by digital caliper for every three days. At the endpoint, mice were then sacrificed, and tumors were surgically excised. The weight and volume of tumors were blinded during quantification, and the tumor images were captured by the camera. The volume calculated formula is V = *π* × Length × Width^2^/6. All studies were approved by the Science and Technology Ethics Committee of School of Public Health, Shandong University.

### Tumor tissues

Mice colorectal tumor tissues were derived from AOM/DSS-induced colorectal C57BL/6 mice models. In brief, 8-week-old male mice were intraperitoneally injected with azoxymethane (AOM) (10 mg/kg body weight) (Sigma-Aldrich). At the 6th day after AOM injection, mice were given 1.5% dextran sulfate sodium (DSS) (MP Biomedicals) in drinking water for 5 days followed by 2 weeks of regular drinking water. DSS treatments were repeated twice, and mice were sacrificed on day 80. Human colorectal tumor tissues and surrounding non-tumor tissues were obtained from First Affiliated Hospital of Shandong First Medical University and Shandong Provincial Qianfoshan Hospital. All the studies were approved by the ethics committee of Shandong University and Shandong Provincial Qianfoshan Hospital, and the Science and Technology Ethics Committee of School of Public Health, Shandong University. All analysis of tumor tissues were performed in a blinded manner, with key findings confirmed by independent replication.

### Immunohistochemistry

Tissue was fixed in 4% PFA and embedded in paraffin. Sections of 5 μm thickness were deparaffinized in xylene and rehydrated. After washing with H_2_O, slides were quenched in 3% H_2_O_2_ in methanol for 15 min. Slides were subsequently rinsed in H_2_O, and antigen retrieval was performed by heating slides to 98 °C in sodium citrate solution for 20 min. Slides were then cooled to room temperature for 1 h, and washed in distilled water for 5 min three times, and blocked with 10% normal goat serum (BOSTER, #AR1009) in PBS for 1 h at room temperature. Subsequently, sections were stained with EHMT2 (Abclonal, #A19288, 1: 400), MBLAC2(Santa Cruz, #SC-398153, 1: 400) at 4 °C overnight. After washing with TBST for 10 min 3 times, sections were incubated with secondary goat anti-mouse/rabbit antibodies for 1 h at room temperature. After washes with TBST for 10 min 3 times, slides were stained with DAB (2,2-diaminobenzidine) (ZSGB-bio, #ZLI-9018) reagent for about 1 min. Slides were then washed in H_2_O and counterstained with Mayer’s hematoxylin (Servicebio, #G1004) for 1 min and Scott’s water solution (Sigma Aldrich) for 1 min. Subsequently, slides were washed, dehydrated in 70% ethanol (1 min), 80% ethanol (1 min), 95% ethanol (1 min), 100% ethanol (2 min), and xylene (3 × 2 min), and mounted using neutral balsam (Solarbio, #G8590). Images were captured by the wide-field Olympus BX53 microscope.

### TCGA-COAD analysis

Transcriptomic gene expression data and clinical data (including survival status, overall survival time, pathological staging, treatment, etc.) for colon adenocarcinoma (COAD) were retrieved from The Cancer Genome Atlas (TCGA) database (https://portal.gdc.cancer.gov/, as of November 2023). Following the exclusion of cases with incomplete clinical records and specific selection of radiation-treated samples, a total of 191 (or 196 for survival analysis) radiotherapy-treated COAD samples (RT-COAD group) and 10 adjacent normal tissue samples (Normal group) were included in the analysis. Survival analysis was performed in R (v 4.4.2) using the R packages “survminer” and “survival” to calculate the optimal cut-off values for EHMT2 and MBLAC2 expression levels. Based on these cutoff values, samples were categorized into high and low expression groups. Kaplan-Meier survival curves were plotted. Gene expression differences between adjacent normal tissue and COAD samples, as well as between adjacent normal tissue and COAD samples of different pathological stages, were analyzed in GraphPad Prism (v9.5.0). Correlation analysis was performed using the ‘corrplot’ R package. Correlation coefficients and *p*-values were calculated using the ‘cor.test’ function with Spearman’s method. Independent-samples *t*-tests were performed for statistical analysis.

### Statistical analysis

All data are presented as mean ± SEM derived from three independent biological replicates unless otherwise delineated. Comparisons between indicated groups were analyzed by two-tailed Student’s *t* test using GraphPad Prism (v9.5.0). Statistical significance is indicated as follows: ns not significant; ^*^P < 0.05, ^**^P < 0.01, ^***^P < 0.001, ^****^P < 0.0001.

## Supplementary information

Supplementary Figures and Legends

Supplementary Table 1-6

Supplementary Table 7

Original blots

## Data Availability

Data from the TCGA-COAD database is available to the public (https://portal.gdc.cancer.gov/). Raw data are available from the corresponding authors upon reasonable request.
