# Correction: The EHMT2-MBLAC2 axis suppresses ribosomal DNA transcription in response to nucleolar DNA damage

**DOI:** 10.1038/s41419-026-08998-2

**Published:** 2026-07-06

**Authors:** Chenyue Wang, Qiutian Lu, Lianbao Cao, Simeng Zeng, Zihan Gao, Yinglong Yang, Xiaowen Liu, Shanshan Gao, Chao Dong

**Affiliations:** 1 Department of Occupational and Environmental Health, School of Public Health, Cheeloo College of Medicine, Shandong University, Jinan, Shandong China; 2 Department of Gynecologic Oncology, Shandong Cancer Hospital and Institute, Shandong First Medical University and Shandong Academy of Medical Sciences, Jinan, Shandong China; 3 Department of Gastroenterology, The First Affiliated Hospital of Shandong First Medical University & Shandong Provincial Qianfoshan Hospital, Jinan, Shandong China; 4 Futaste Pharmaceutical Co., Ltd., Dezhou, Shandong China

**Keywords:** DNA damage and repair, Molecular biology

Correction to: *Cell Death & Disease* 10.1038/s41419-026-08616-1, published online 18 March 2026

We noticed a discrepancy regarding the author affiliations in the online version, which do not match the final proof we approved. It appears that the affiliations for our co-corresponding author, Shanshan Gao, were not updated to match our final submitted proof. Currently, they are listed as follows:

3 Futaste Pharmaceutical Co. Ltd., Dezhou, Shandong, China.

4 Present address: Department of Gastroenterology, The First Affiliated Hospital of Shandong First Medical University & Shandong Provincial Qianfoshan Hospital, Jinan, Shandong, China.

It should be listed as :

3 Department of Gastroenterology, The First Affiliated Hospital of Shandong First Medical University & Shandong Provincial Qianfoshan Hospital, Jinan, Shandong, China.

4 Futaste Pharmaceutical Co. Ltd., Dezhou, Shandong, China.

The original article has been corrected.

