# Supplementary material for: The EHMT2-MBLAC2 axis suppresses ribosomal DNA transcription in response to nucleolar DNA damage
Source: Cell Death Dis. 2026 Mar 18;17(1):320. doi: 10.1038/s41419-026-08616-1 (PMC13039405; doi:10.1038/s41419-026-08616-1)
Supplement: Supplementary file 1 — Supplementary Figures and Legends [file 41419_2026_8616_MOESM1_ESM.pdf]

Supplementary Figure S1

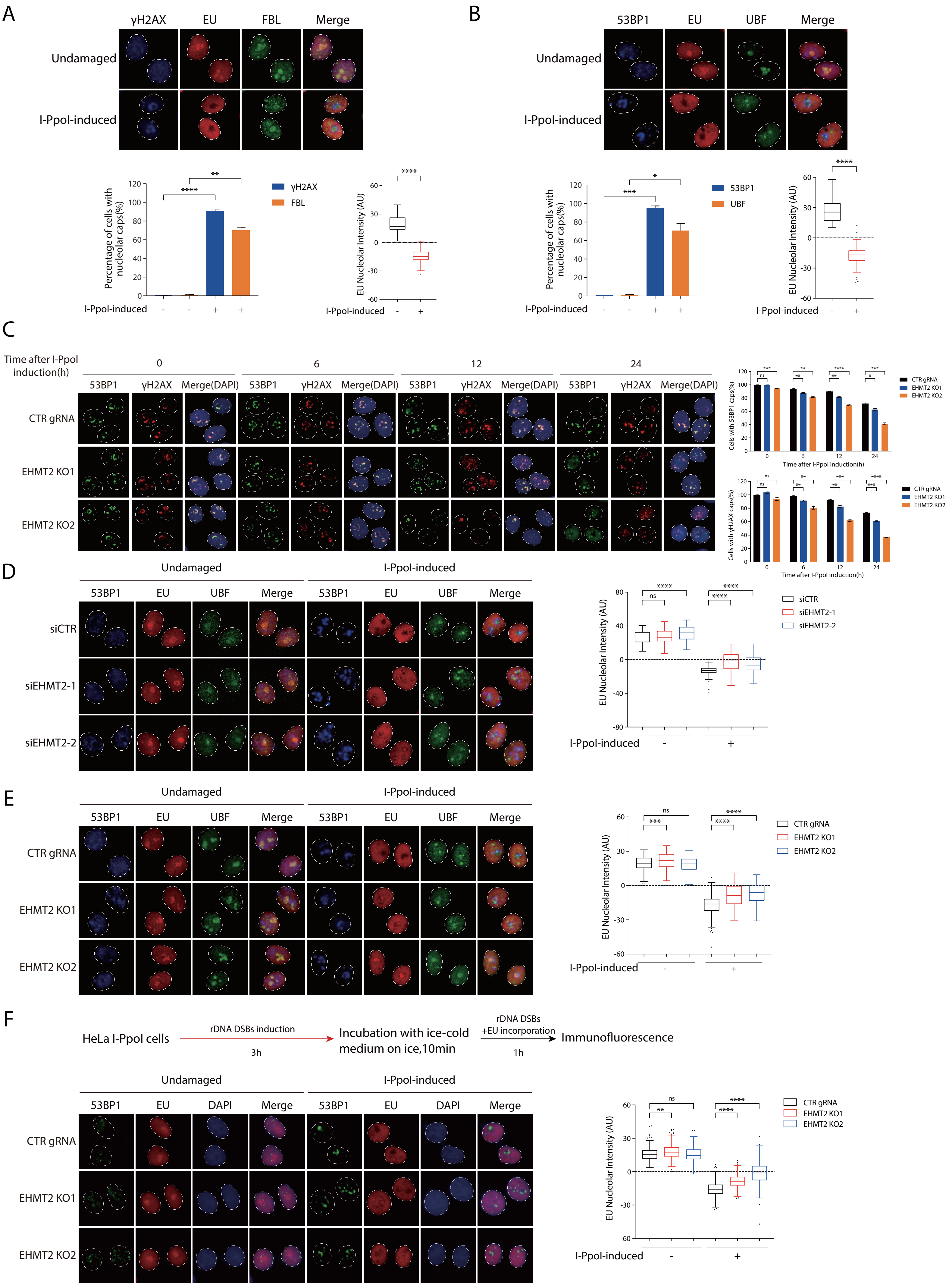

Supplementary Figure S2

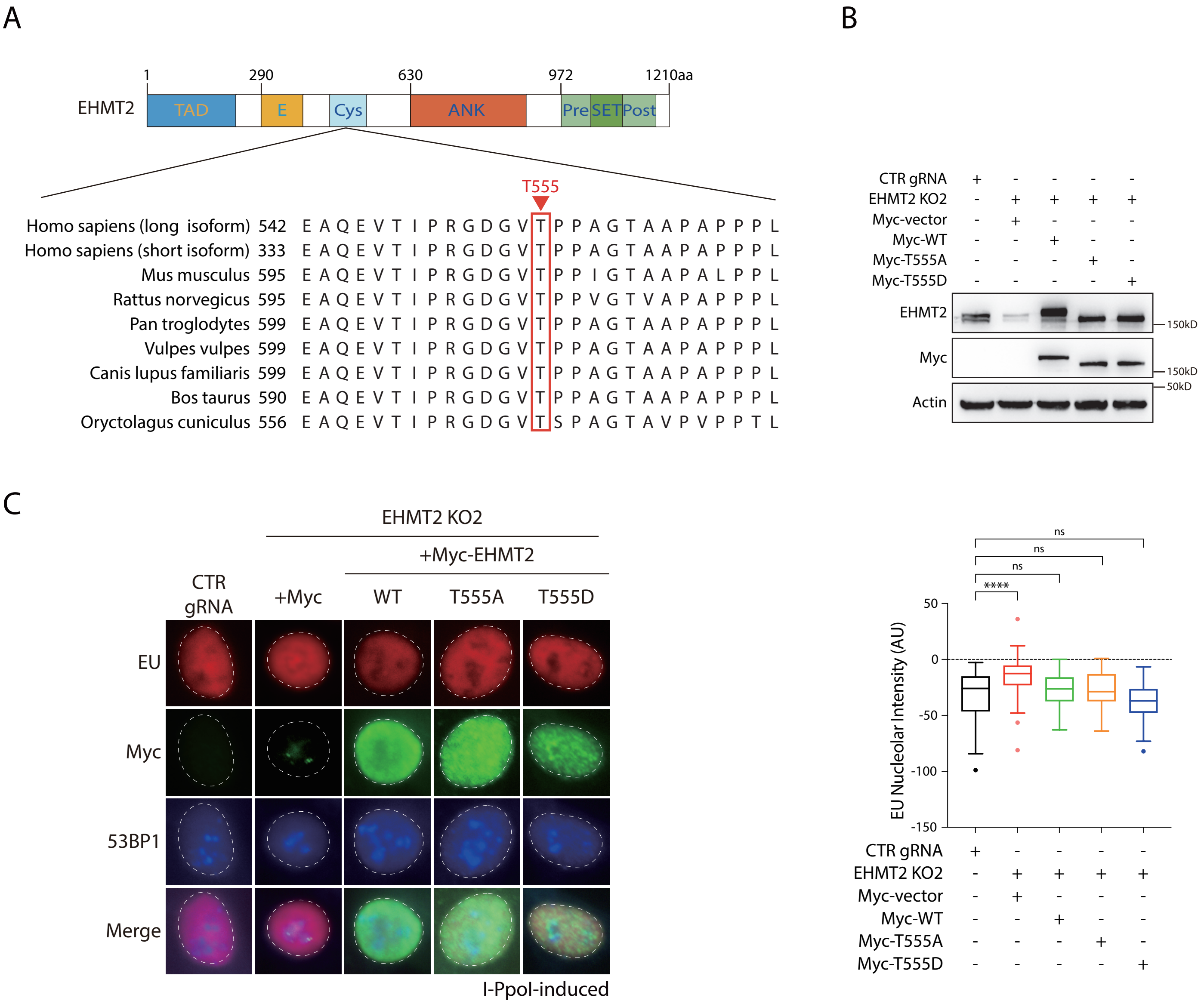

Supplementary Figure S3

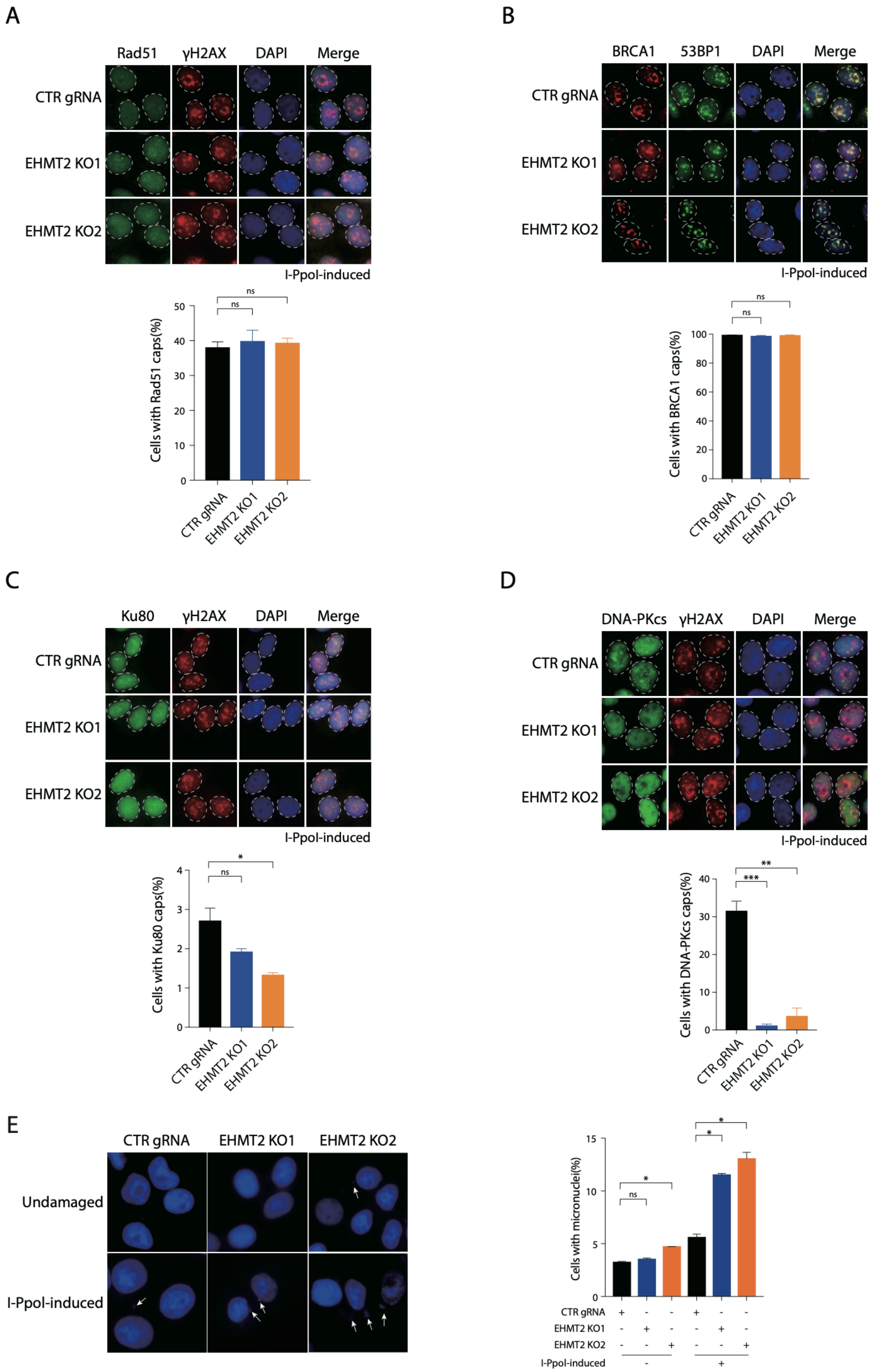

Supplementary Figure S4

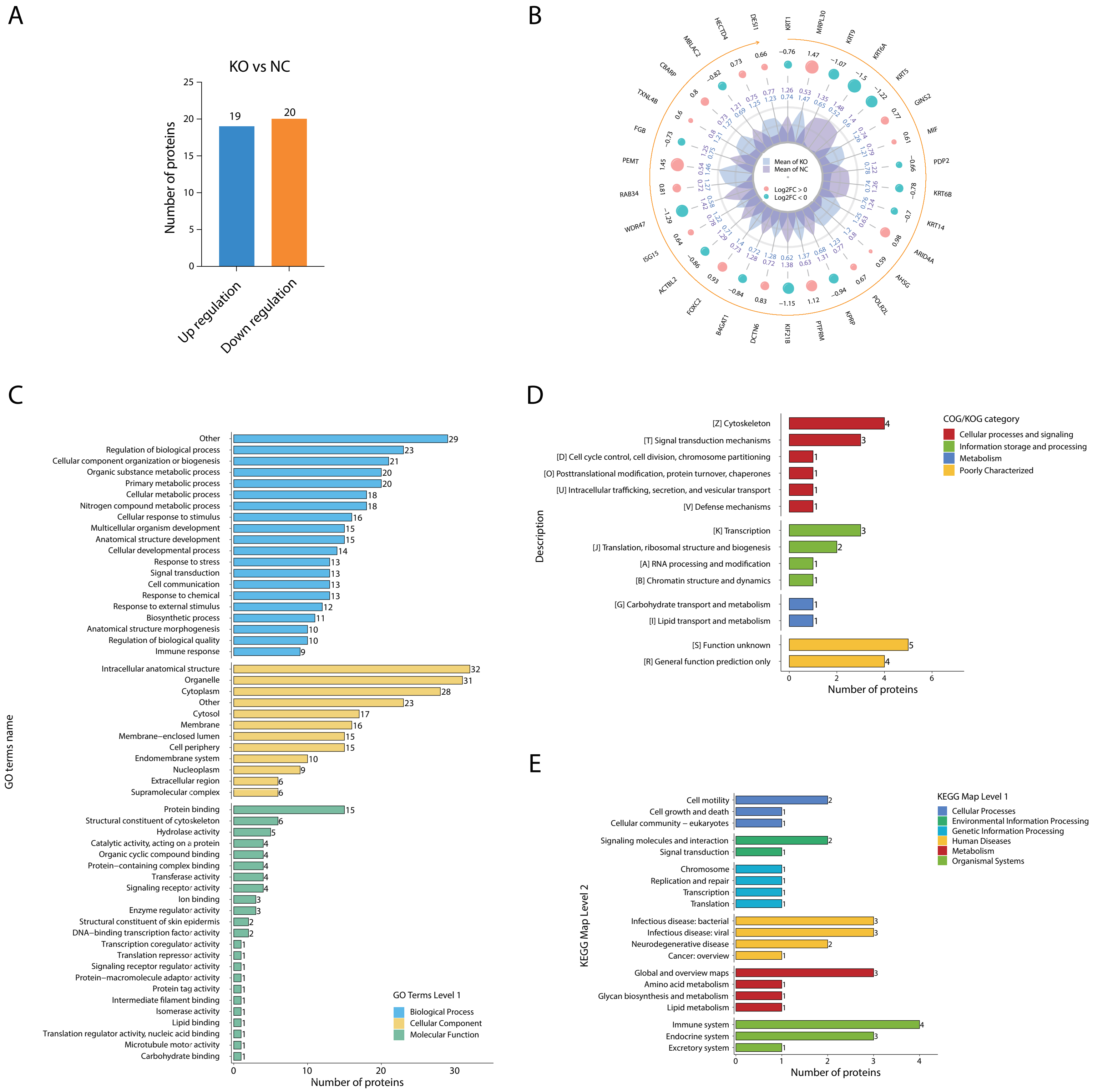

Supplementary Figure S5

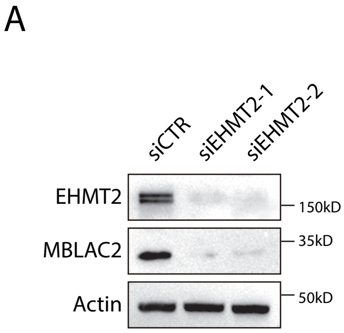

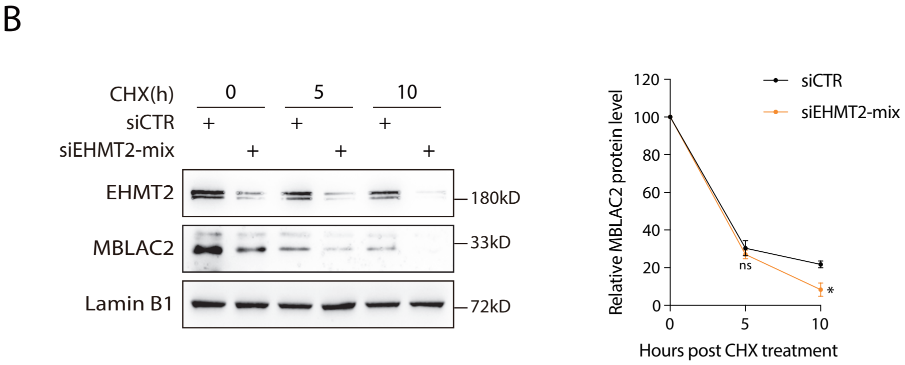

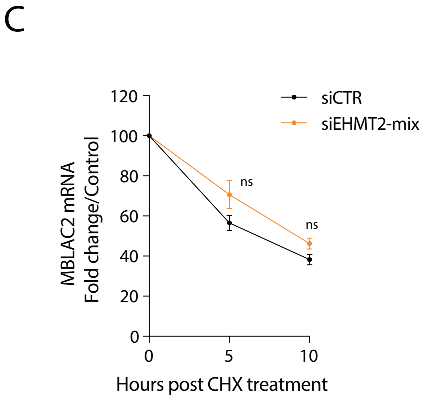

Supplementary Figure S6

A

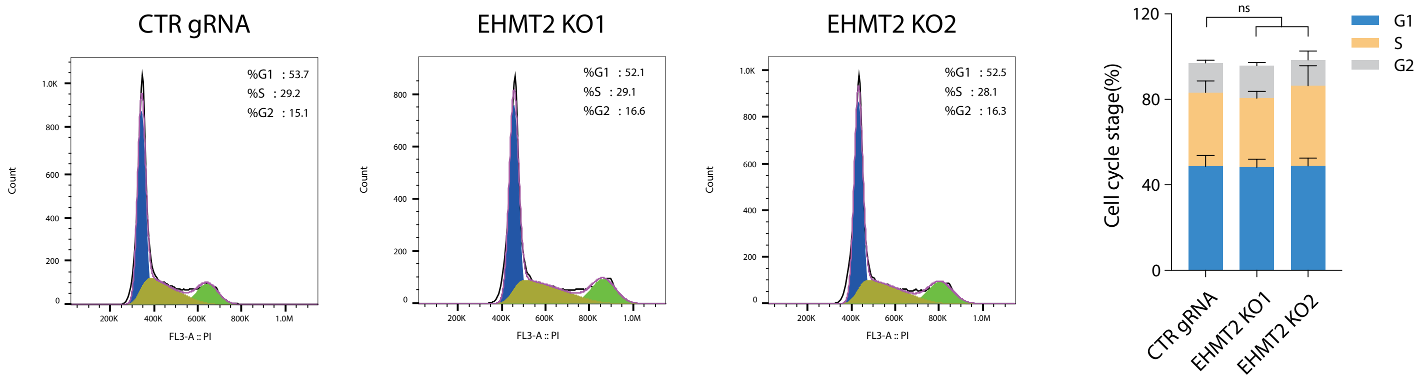

B

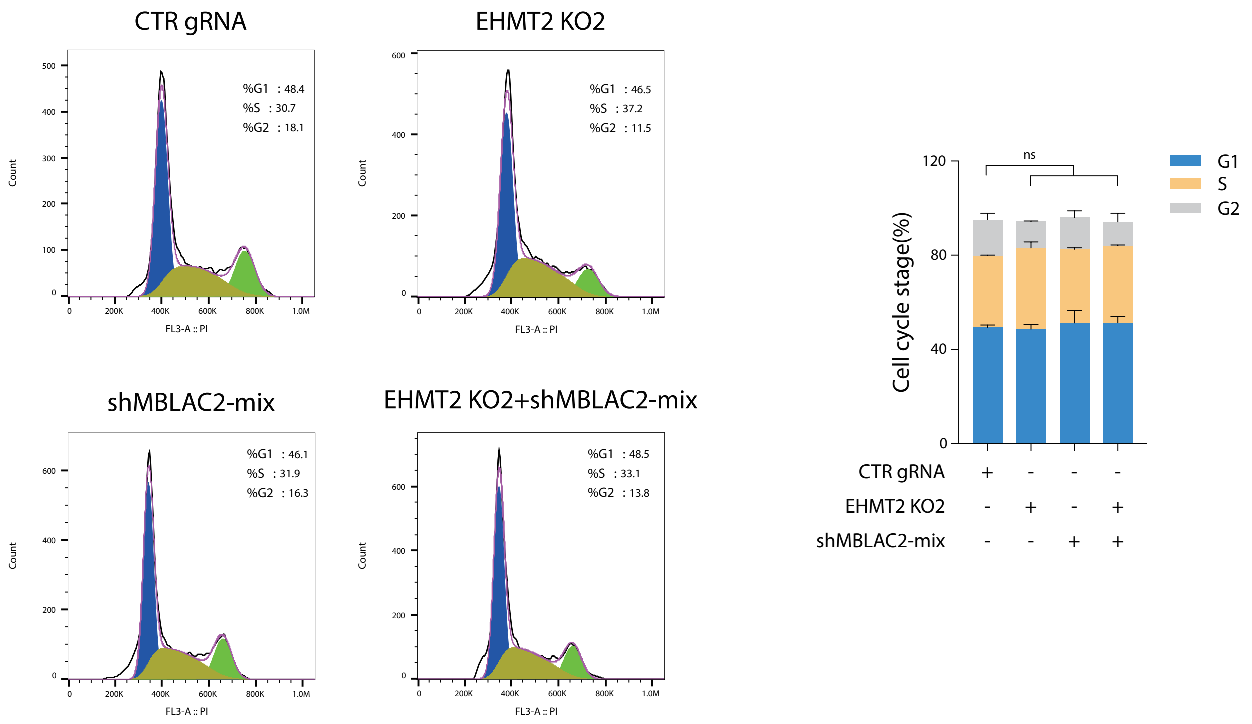

Supplementary Figure S7

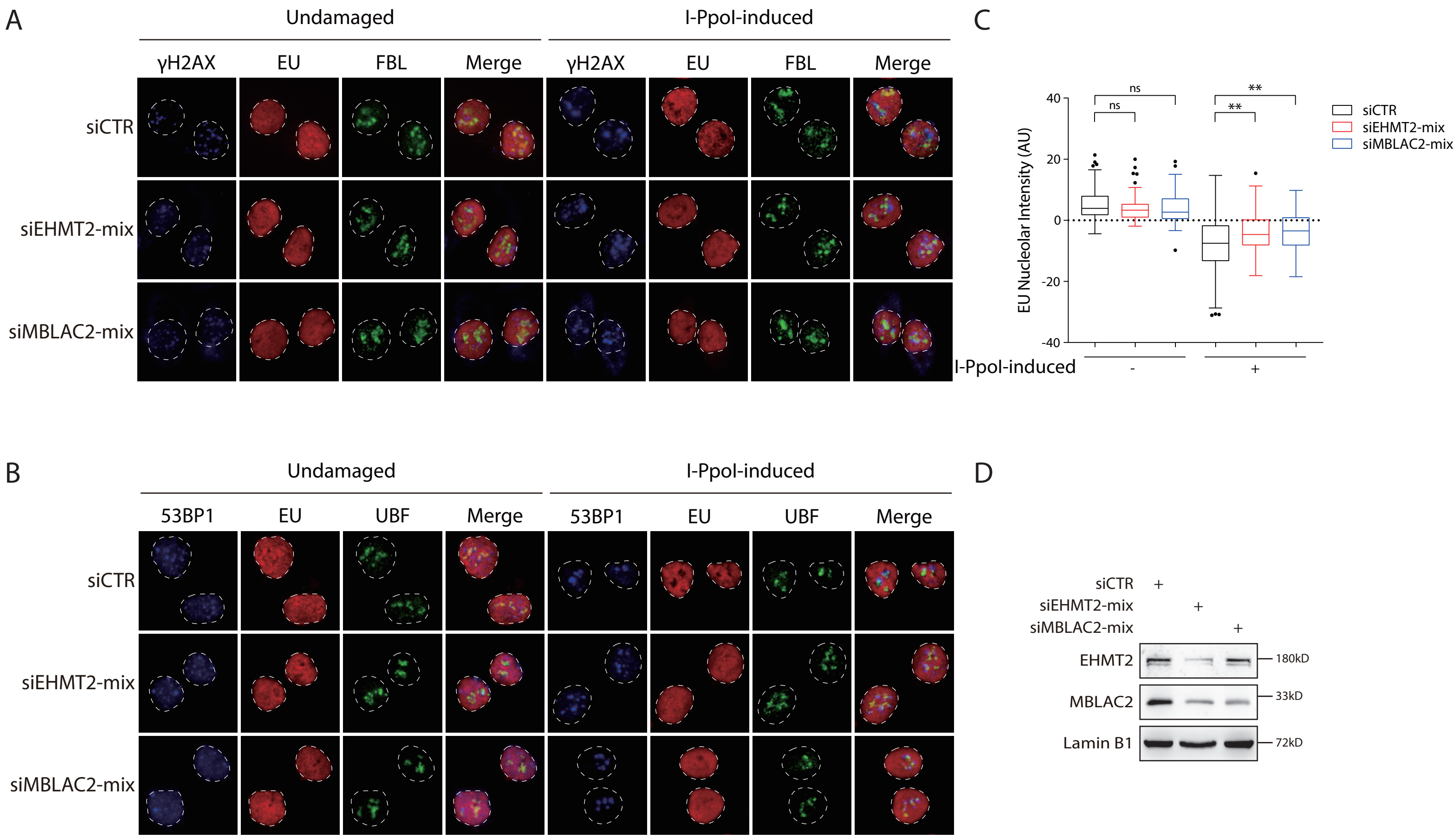

Supplementary Figure S8

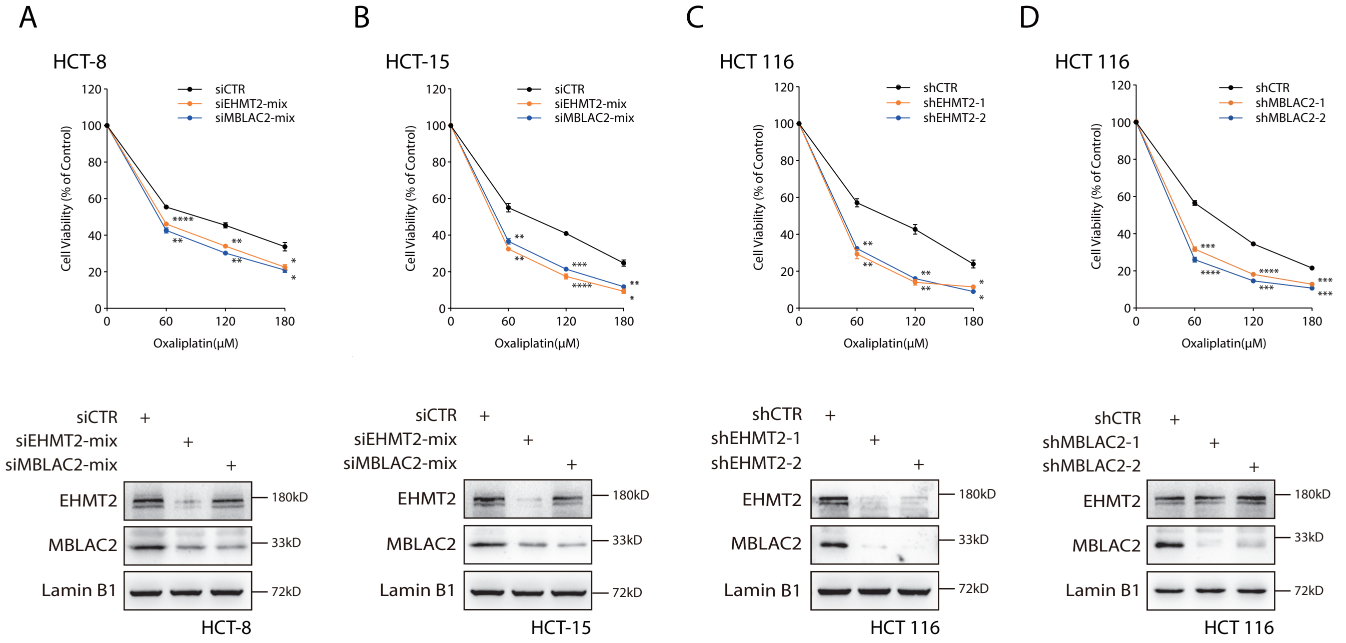

Supplementary Figure S9

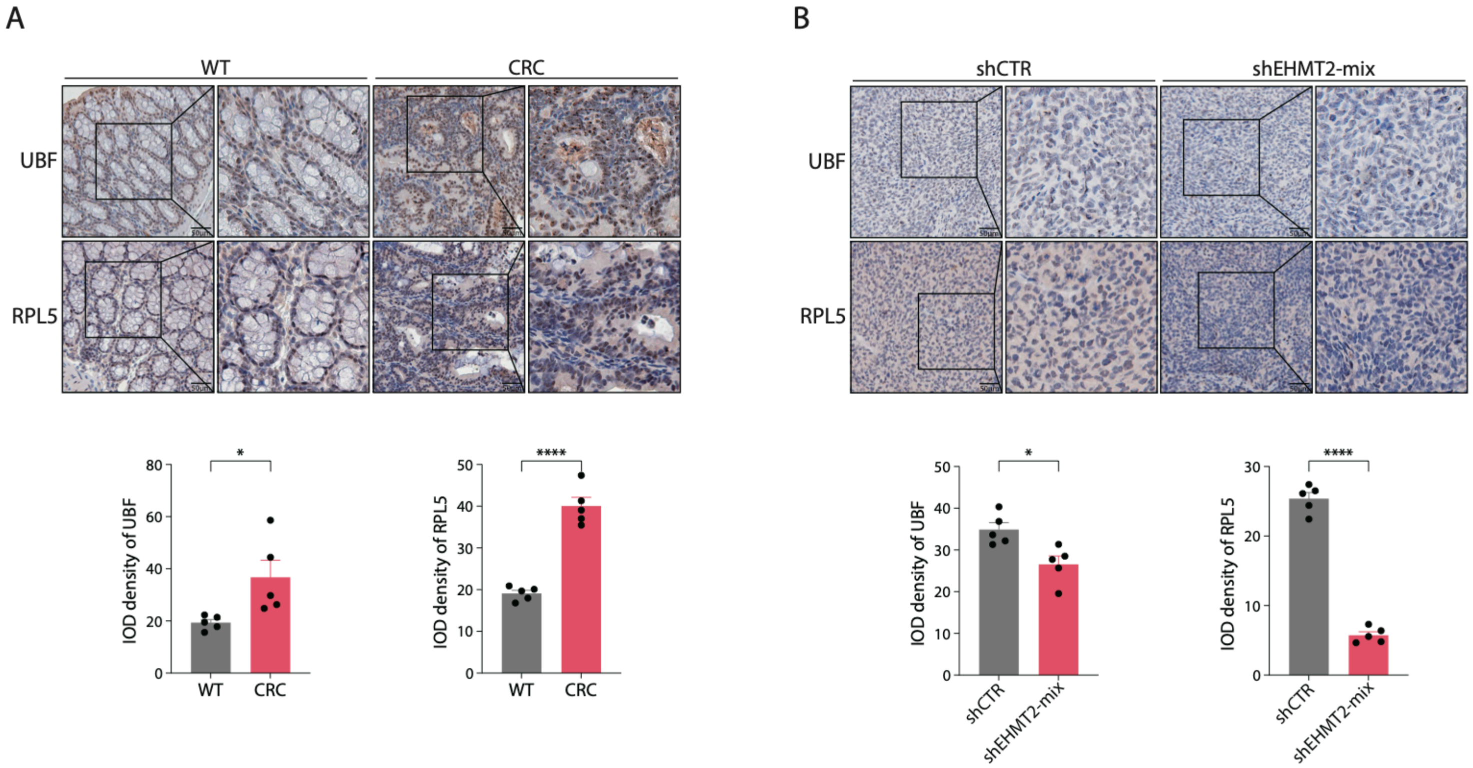

Supplementary Figure S10

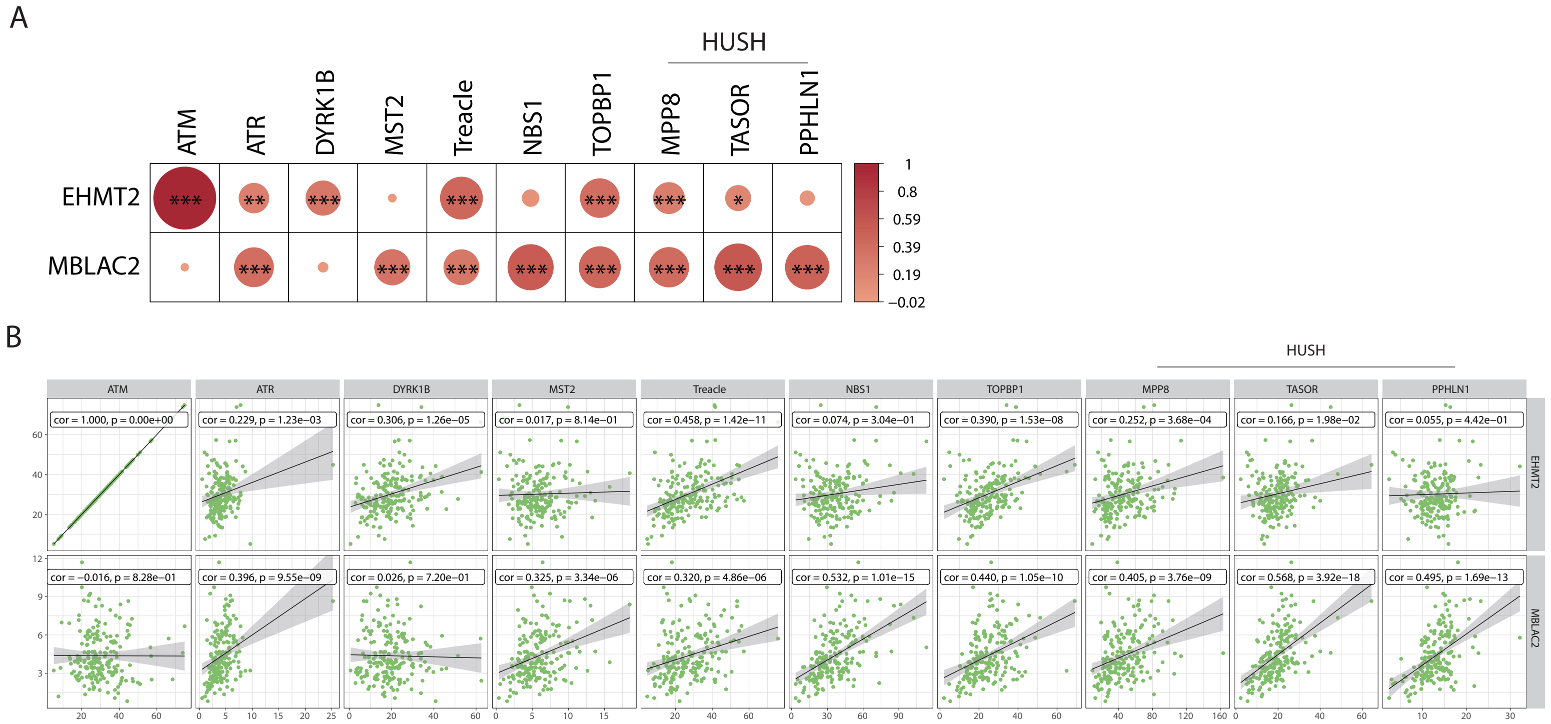

Supplementary Figure Legends

Supplementary Figure S1. Validation of inducible I-*Ppo*I cells.

(A&B) rDNA DSBs in HeLa I-*Ppo*I cells were induced with 1 µM Shield-1 and 2 µM 4-OHT for 4 h before fixation. Cells were subsequently processed for immunofluorescence with anti-γH2AX and anti-FBL (A) or anti-53BP1 and anti-UBF (B) antibodies. Nucleolar EU intensity was measured by EU incorporation assays. Percentage of cells with nucleolar caps and nucleolar EU intensity was analyzed. Data were derived from three independent experiments. **(C)** HeLa I-*Ppo*I cells transduced with control gRNA (CTR gRNA) and EHMT2 gRNAs (EHMT2 KO1 and EHMT2 KO2) were induced for rDNA DSBs for 4 h. After washing with PBS twice, cells were allowed to grow for 0, 6, 12, or 24 h. Cells were subsequently processed for immunofluorescence with anti-53BP1 and anti-γH2AX antibodies. Nuclei were counterstained with DAPI. At least 700 cells were analyzed and data were derived from two independent experiments. **(D)** Analysis of nucleolar transcription activity by 5-Ethynyl Uridine (EU) incorporation assay in EHMT2-deficient HeLa I-*Ppo*I cells following rDNA DSBs induction. Cells transfected with control (siCTR) or two independent EHMT2-targeting siRNAs were induced for rDNA DSBs for 4 h. Cells were subsequently cultured in medium supplemented with EU for 1 h before fixation. Fixed cells were labelled with EU, 53BP1 and UBF. Quantification of relative EU nucleolar intensity from three independent experiments is shown in Tukey boxplots. **(E)** HeLa I-*Ppo*I cells transduced with control gRNA (CTR gRNA) and two EHMT2 gRNAs (EHMT2 KO1 and EHMT2 KO2) were subjected to EU incorporation assay as described in (C). Quantification of relative EU nucleolar intensity from three independent experiments is shown in Tukey boxplots. **(F)** EU incorporation assay was assessed in HeLa I-*Ppo*I cells transduced with control gRNA (CTR gRNA) or one of the two independent EHMT2-targeting gRNAs (EHMT2 KO1 and EHMT2 KO2) following the workflow. At least 200 cells exhibiting well-circumscribed nucleoli were quantitatively assessed across two independent experiments. Quantification of relative EU nucleolar intensity is shown in Tukey boxplots. Bars represent mean ± SEM; ns, not significant; $^{*}P < 0.05$; $^{**}P < 0.01$; $^{***}P < 0.001$; $^{****}P < 0.0001$.

Supplementary Figure S2. EHMT2 mediates rDNA DSB-induced transcriptional silencing independent of its T555 phosphorylation.

(A) Schematic illustration of EHMT2 protein domains and the DYRK1B-phosphorylated site at T555. T555 denotes T346 in the short isoform of EHMT2. **(B)** Immunoblot analysis was performed in HeLa I-*Ppo*I EHMT2 KO2 cells reconstituted with Myc-tagged EHMT2 WT and its phosphorylation site mutants (T555D and T555A). **(C)** Cells described in (B) were subjected to EU incorporation assay after I-*Ppo*I induction. Cells were labelled with anti-53BP1 antibodies to denote nucleolar caps. Nuclei were counterstained with DAPI. Quantification of EU nucleolar intensity was analyzed and data were derived from three independent experiments. Bars represent mean ± SEM; ns, not significant; $^{****}P<0.0001$.

Supplementary Figure S3. Nucleolar caps formation of DSBs repair proteins in EHMT2-deficient cells following rDNA DSB induction.

(A-D) HeLa I-*Ppo*I cells transduced with control gRNA (CTR gRNA) and EHMT2 gRNAs (EHMT2 KO1 and EHMT2 KO2) were processed for immunofluorescence using Rad51 (A), BRCA1 (B),

Ku80 (C), DNA-PKcs (D) with 53BP1 or γH2AX antibodies. Nuclei were counterstained with DAPI. Quantification of cells with the indicated nucleolar caps was analyzed and data were derived from three independent experiments. **(E)** Representative images depict the micronuclei derived from undamaged and I-*Ppo*I induced EHMT2 KO cells. Quantification of the percentage of cells with micronuclei as shown. Data derived from three independent experiments were quantified. White arrowheads delineate the sites of micronuclei. Bars represent mean ± SEM; ns, not significant; $^{*}P < 0.05$; $^{**}P < 0.01$; $^{***}P < 0.001$.

Supplementary Figure S4. Proteomic analysis of EHMT2 substrates in HeLa I-*Ppo*I cells following rDNA DSBs.

(A) Histogram shows the number of significant upregulation and downregulation proteins in EHMT2 KO vs EHMT2 control cells after rDNA DSBs. **(B)** The top 30 differentially expressed proteins is shown in a radar chart to display their relative expression levels. The outermost ring indicates the names of the differentially expressed proteins. Proteins are arranged clockwise along the orange arrow, sorted by either ascending *P*-value or coefficient of variation (CV) or descending absolute value of the Log2-transformed fold change. The second ring represents Log2-transformed ratio values, while pink denotes upregulation, light blue indicates downregulation, respectively. The size of the points corresponds to the magnitude of the fold change. The innermost ring indicates the average quantitative levels of the two categories. Sharp peaks suggest the significantly elevated expression levels of the proteins. **(C)** Gene Ontology (GO) enrichment analysis of the differentially expressed proteins. **(D)** Functional annotation of the differentially expressed proteins was conducted using the Clusters of Orthologous Groups (COG) and Eukaryotic Orthologous Groups (KOG) databases. **(E)** Pathway enrichment analysis of the differentially expressed proteins was performed using the Kyoto Encyclopedia of Genes and Genomes (KEGG) database was displayed.

Supplementary Figure S5. The decreased expression of MBLAC2 in EHMT2-deficient cells.

(A) Immunoblot of MBLAC2 and EHMT2 in the HeLa I-*Ppo*I cells transfected with siRNAs targeting EHMT2. **(B)** HeLa I-*Ppo*I cells transfected with control (siCTR) and siRNA-mix (siEHMT2-1 and siEHMT2-2) were treated with cycloheximide for 0, 5, and 10 h. The protein expression of MBLAC2 and EHMT2 in HeLa I-*Ppo*I cells was examined by immunoblot. The relative MBLAC2 protein level was measured. Data were derived from three independent experiments. **(C)** HeLa I-*Ppo*I cells were transfected with control (siCTR) and siRNA-mix (siEHMT2-1 and siEHMT2-2). Fold change of MBLAC2 mRNA in I-*Ppo*I cells treated with cycloheximide was determined by RT-qPCR. Quantification of MBLAC2 mRNA fold change was from three independent experiments. Bars represent mean ± SEM; ns, not significant; $^{*}P < 0.05$.

Supplementary Figure S6. Cell cycle analysis of EHMT2- and MBLAC2-deficient cells.

(A) HeLa I-*Ppo*I cells transduced with control gRNA (CTR gRNA) and EHMT2 gRNAs (EHMT2 KO1 and EHMT2 KO2) were subjected to flow cytometry analysis. Cell cycle stage was quantified. **(B)** HeLa I-*Ppo*I cells transduced with EHMT2 gRNA2 or combined with MBLAC2-targeting shRNAs were subjected to flow cytometry analysis. Cell cycle stage was plotted and quantified. Bars represent mean ± SEM; ns, not significant.

Supplementary Figure S7. Nucleolar EU intensity in EHMT2- or MBLAC2-deficient HCT 116 I-PpoI cells upon rDNA DSB induction.

(A) Analysis of nucleolar transcription activity by 5-Ethynyl Uridine (EU) incorporation assay in EHMT2-deficient HCT 116 I-*Ppo*I cells following rDNA DSBs induction. Cells transfected with control (siCTR), EHMT2-targeting siRNA-mix (siEHMT2-1 and siEHMT2-2) or MBLAC2-targeted siRNA-mix (siMBLAC2-1, siMBLAC2-2 and siMBLAC2-3) were induced for rDNA DSBs for 4 h. Cells were subsequently cultured in medium supplemented with EU for 1 h before fixation. Fixed cells were labelled with EU, γH2AX and Fibrillarin (FBL). **(B)** HCT 116 I-*Ppo*I cells were subjected to EU incorporation assay as described in (A). Fixed cells were labelled with EU, 53BP1 and UBF. **(C)** Quantification of relative EU nucleolar intensity from two independent experiments is shown in Tukey boxplots. **(D)** The knockdown efficiency of EHMT2 and MBLAC2 was examined by immunoblotting. Bars represent mean ± SEM; ns, not significant; $^{**}P < 0.01$.

Supplementary Figure S8. EHMT2 and MBLAC2 promote the survival of colon cancer cells under ribosome biogenesis stress.

(A) HCT-8 cells transfected with control siRNA (siCTR), EHMT2-targeting siRNA mix (siEHMT2-1 and siEHMT2-2) or MBLAC2-targeting siRNA mix (siMBLAC2-1 and siMBLAC2-2) were treated with the indicated concentrations of oxaliplatin for 6 h. Cell viability was measured by CCK-8 assay and is presented as a percentage of the untreated control (upper panel). Immunoblot analysis validating the knockdown efficiency of EHMT2 and MBLAC2 in HCT-8 cells is shown below (lower panel). **(B)** HCT-15 cells transfected with the indicated siRNAs were subjected to CCK-8 assay following the same procedures as depicted in (A). The corresponding immunoblot validating EHMT2 and MBLAC2 knockdown efficiency in HCT-15 cells was presented. **(C)** HCT 116 cells transduced with control shRNA (shCTR) or two independent shRNAs targeting EHMT2 (shEHMT2-1 and shEHMT2-2) were treated with oxaliplatin, and cell viability was assessed (upper panel). The lower panel shows immunoblot analysis verifying EHMT2 knockdown efficiency in HCT 116 cells. **(D)** HCT 116 cells transduced with control shRNA (shCTR) or two independent shRNAs targeting MBLAC2 (shMBLAC2-1 and shMBLAC2-2) were treated and analyzed for cell viability (upper panel). Immunoblot analysis confirming MBLAC2 knockdown in these cells was displayed (lower panel). Data in all panels are derived from three independent experiments and are presented as mean ± SEM; ns, not significant; $^{*}P < 0.05$; $^{**}P < 0.01$; $^{***}P < 0.001$; $^{****}P < 0.0001$.

Supplementary Figure S9. IHC staining of UBF and RPL5 in mouse CRC and nude mice tumor sections.

(A) Representative IHC images stained with UBF and RPL5 in colon or colon tumors from WT and CRC tissues, respectively. Scale bar=50 μm. The enlarged pictures show the details of UBF and RPL5 staining. Integrated optical density (IOD) of UBF and RPL5 were quantified based on five IHC images derived from three independent experiments. **(B)** Representative IHC images of UBF- and RPL5-stained tumor sections derived from excised tumors of nude mice subcutaneously injected with either control (shCTR) or shRNA-mix (shEHMT2-1 and shEHMT2-2) transduced HCT 116 cells. Scale bar=50 μm. The enlarged pictures show the details of staining. Quantification of IOD of UBF and RPL5 from five representative IHC images. Bars

represent mean ± SEM; $^{*}P < 0.05$; $^{****}P < 0.0001$.

Supplementary Figure S10. Correlation analysis of EHMT2 and MBLAC2 with transcription factors in RT-COAD datasets.

(A&B) Heatmap (A) and scatter plots (B) showing the correlation between the expression of EHMT2 or MBLAC2 and known regulators of rDNA DSB-dependent silencing in RT-COAD samples. $^{*}P<0.05$, $^{**}P<0.01$, $^{***}P<0.001$.
