# Supplementary material for: The EHMT2-MBLAC2 axis suppresses ribosomal DNA transcription in response to nucleolar DNA damage
Source: Cell Death Dis. 2026 Mar 18;17(1):320. doi: 10.1038/s41419-026-08616-1 (PMC13039405; doi:10.1038/s41419-026-08616-1)
Supplement: Supplementary file 2 — Supplementary Table 1-6 [file 41419_2026_8616_MOESM2_ESM.pdf]

Supplementary Table 1. Plasmids used in this study

Plasmids	Source	Identifier
pLVX-PTuner-I-PpoI	Gift from Michael S.Y. Huen	N/A
LentiCRISRR V2 vector	Addgene	Plasmid #52961
pMH-MYC	Addgene	Plasmid #101765
pMH-SFB	Addgene	Plasmid #99391
psPAX2	Addgene	Plasmid #12260
PMD2.G	Addgene	Plasmid #12259
pLVpuro-CMV-N-mCherry	Addgene	Plasmid #123221
pLVpuro-CMV-N-EGFP	Addgene	Plasmid #122848
pLKO.1 puro	Addgene	Plasmid #8453
pLVpuro-CMV-N-EGFP-EHMT2	This study	N/A
pLVpuro-CMV-N-EGFP-MBLAC2	This study	N/A
pMH-MYC-EHMT2	This study	N/A
pMH-SFB-MBLAC2	This study	N/A
Myc-EHMT1-D1	This study	N/A
Myc-EHMT1-D2	This study	N/A
Myc-EHMT1-D3	This study	N/A
Myc-EHMT1-D4	This study	N/A
Myc-EHMT1-D5	This study	N/A
Myc-EHMT1-D6	This study	N/A

Supplementary Table 2. Sequences of gRNAs used in this study

gRNAs	Sequences
Non-targeting gRNA (CTR gRNA)	GTATTACTGATATTGGTGGG
EHMT2 gRNA#1	GGGTCACTTCTCCTGAACGC
EHMT2 gRNA#2	CACGCGCTCATCCACAGAGT

Supplementary Table 3. Sequences of shRNAs used in this study

shRNAs	Sequences
N4BP1-shRNA1	CACGCCTTGTTCTCCACTGA
N4BP1-shRNA2	AGATATAAAAGAAACTACTG
N4BP1-shRNA3	CGTGATCCTAATGTCACAGA
MBLAC2-shRNA1	CTTACATGACAAAGACCGAA
MBLAC2-shRNA2	CTTCTGGATTCAAGAACGTTT
MBLAC2-shRNA3	CAAGTTTAGCTCTACGTGTAA
KRT5-shRNA1	GCCGGTAGTGGATTTGGTTTC
KRT5-shRNA2	GCACTGATGGATGAGATTAAC
KRT5-shRNA3	GCATGTCTCTGACACCTCAGT
WDR47-shRNA1	AGAAACAGTGAATGTAAAAG
WDR47-shRNA2	GCTAATACTTGATGGTCAATG
WDR47-shRNA3	GGAGAAGAAATTACAGAAAGC
KRT6A-shRNA1	CCAGCAGGAAGAGCTATA
KRT6A-shRNA2	GAGGAGATTGCTCAGAGAAGC
KRT6A-shRNA3	GAACAAGGTTGAACTGCAAGC
EHMT2-shRNA1	GCUCUAACUGAACAACUAA
EHMT2-shRNA2	CGCUGAUUUUCGAGUGUAA

Supplementary Table 4. Sequences of siRNAs used in this study

siRNAs	Sequences
Non-targeting siRNA (CTR siRNA)	UUCUCCGAACGUGUCACGUTT
EHMT2-siRNA1	GCUCUAACUGAACAACUAA
EHMT2-siRNA2	CGCUGAUUUUCGAGUGUAA
MBLAC2-siRNA1	GTATTTGCTTACATGACAA
MBLAC2-siRNA2	GGACGTGGTGATCGATACA
MBLAC2-siRNA3	GCGATCTCTTGCAAGTTTA

Supplementary Table 5. Sequences of primers used in RT-qPCR

Primers	Sequences
GAPDH	F: 5'- TGCACCACCAACTGCTTAGC -3'
	R: 5'- GGCATGGACTGTGGTCATGAG -3'
45S pre-rRNA	F: 5'- CCGCGCTCTACCTTACCTAC -3'
	R 5'- GAGCGACCAAAGGAACCATA -3'
N4BP1	F: 5'- TCTGAAGAAAGGCATACGAAGAAGC -3'
	R 5'- CAGCAGAAGAATCAGATAGGTCAGC -3'
MBLAC2	F: 5'- TTTGCTTACATGACAAAGACCGA -3'
	R 5'- GTTAGAAGCCAATCGAAAAAGCC -3'
WDR47	F: 5'- CCAGTGATGTTCGCTCTGTTCG -3'
	R 5'- CATGATAGGAAGCTGCTTGGTGAG -3'
KRT5	F: 5'- CATGTCTCGCCAGTCAAGTGTG -3'
	R 5'- TGCCGAAGCCACCACCAC -3'
KRT6A	F: 5'- ACCATCTGAGCACCCATTGC -3'
	R 5'- TCTGAAATAAGCCCCTGGCA -3'

Supplementary Table 6. Sequences of primers used for the measurement of rDNA copy number in this study

Primers	Sequences
GAPDH	F: 5'- CACGCCTTGTTCTCCACTGA -3'
	R: 5'- AGATATAAAAGAAACTACTG -3'
18S rDNA-Primer-1	F: 5'-GTTGGTGGAGCGATTTGTCT-3'
	R 5'-CGCTGAGCCAGTCAGTGTAG-3'
18S rDNA-Primer-2	F: 5'-ACCACATCCAAGGAAGGCAG-3'
	R: 5'-CGCTATTGGAGCTGGAATTAC-3'
28S rDNA Primer-1	F: 5'-ACGCGATGTGATTTCTGCCC-3'
	R: 5'-TCTTCTTTCCCCGCTGATTCC-3'
28S rDNA Primer-2	F: 5'-TGGAGCAGAAGGGCAAAAGC-3'
	R: 5'-TAGGAAGAGCCGACATCGAAGG-3'
28S rDNA Primer-3	F: 5'-CTTCGATGTCGGCTCTTCCTA-3'
	R: 5'-CCTCAGCCAAGCACATACACC-3'
