# Supplementary material for: The EHMT2-MBLAC2 axis suppresses ribosomal DNA transcription in response to nucleolar DNA damage
Source: Cell Death Dis. 2026 Mar 18;17(1):320. doi: 10.1038/s41419-026-08616-1 (PMC13039405; doi:10.1038/s41419-026-08616-1)

Figure 1E

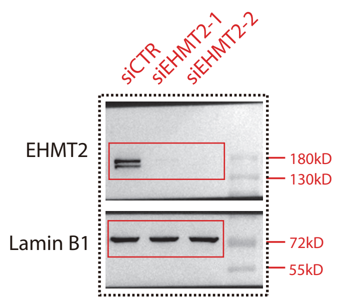

Figure 1F

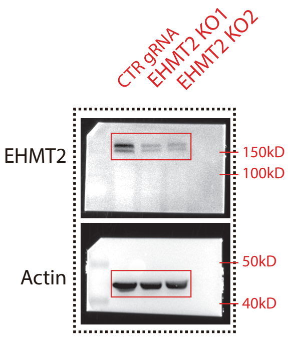

Figure 2D

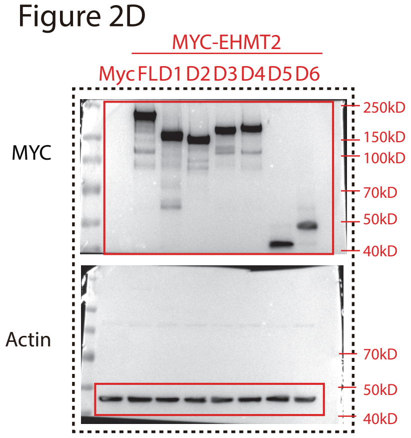

Figure 2F

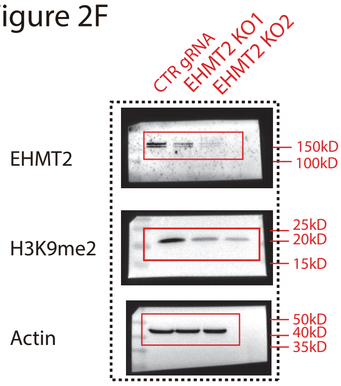

Figure 4G

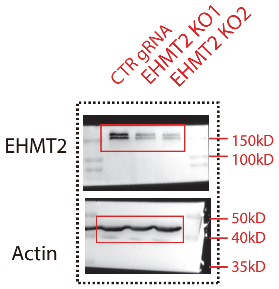

Figure 6C

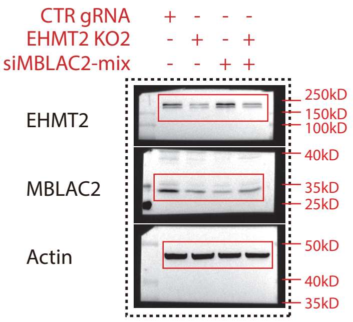

Figure 6D

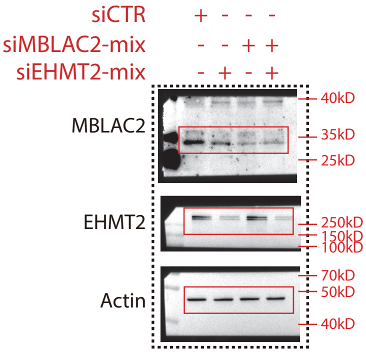

Figure 6E

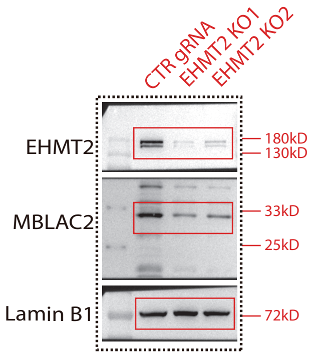

Figure 6F

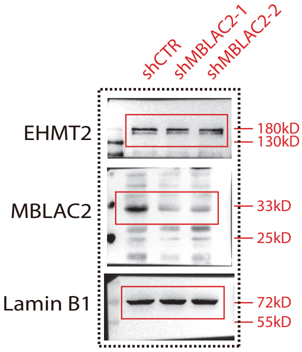

Figure 6G

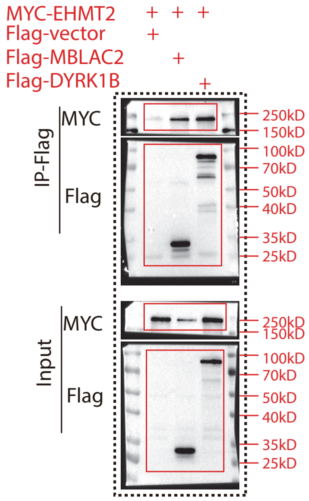

Figure 6H

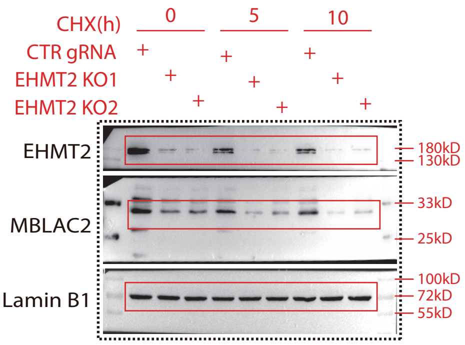

Figure 7B

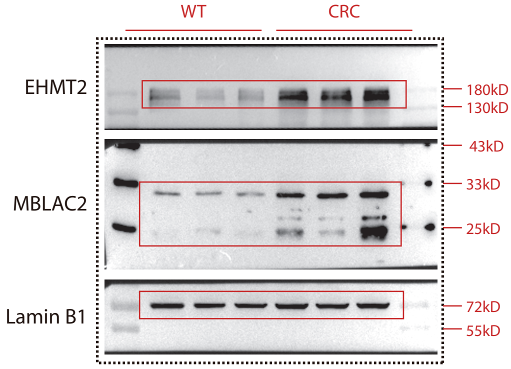

Figure 7E

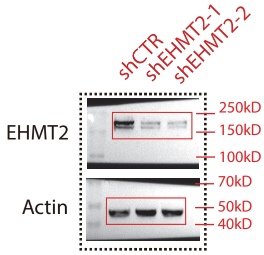

Supplementary Figure 2B

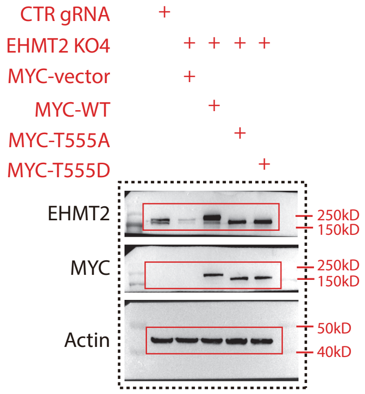

Supplementary Figure 5A

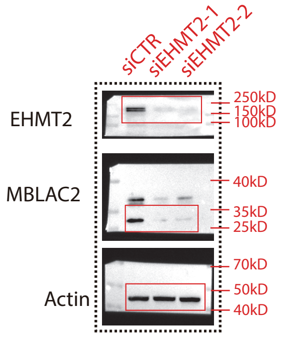

Supplementary Figure 5B

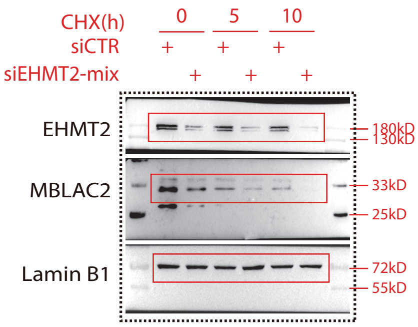

Supplementary Figure 7D

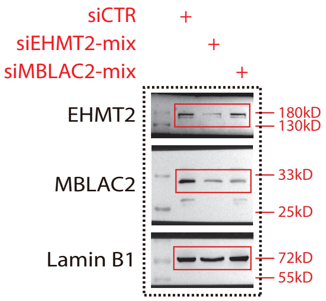

Supplementary Figure 8A

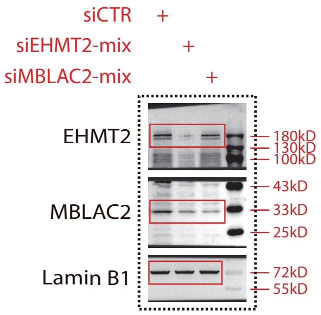

Supplementary Figure 8B

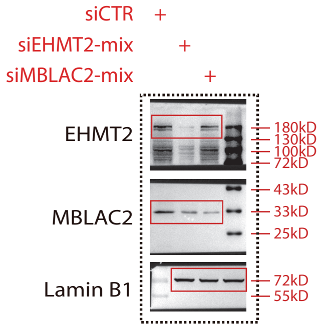

Supplementary Figure 8C

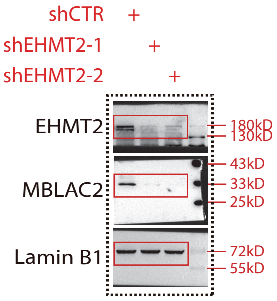

Supplementary Figure 8D

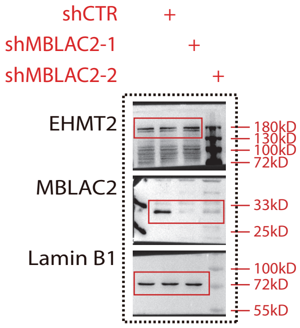

Supplement: Supplementary file 4 — Original blots [file 41419_2026_8616_MOESM4_ESM.pdf]
